# Tau induces inflammasome activation and microgliosis through acetylating NLRP3

**DOI:** 10.1002/ctm2.1623

**Published:** 2024-03-15

**Authors:** Lun Zhang, Yongkang Gai, Yushuang Liu, Dongli Meng, Yi Zeng, Yong Luo, Huiliang Zhang, Zhuoqun Wang, Mengzhe Yang, Yunfan Li, Yi Liu, Yiwen Lai, Jiayu Yang, Gang Wu, Yu Chen, Jingtan Zhu, Shaojun Liu, Tingting Yu, Ji Zeng, Jianzhi Wang, Dan Zhu, Xiaochuan Wang, Xiaoli Lan, Rong Liu

**Affiliations:** ^1^ Department of Pathophysiology School of Basic Medicine, Key Laboratory of Education Ministry of China/Hubei Province for Neurological Disorders, Tongji Medical College, Huazhong University of Science and Technology Wuhan China; ^2^ Department of Clinical Laboratory Wuhan Fourth Hospital Wuhan China; ^3^ Department of Nuclear Medicine Hubei Province Key Laboratory of Molecular Imaging, Union Hospital, Tongji Medical College, Huazhong University of Science and Technology Wuhan China; ^4^ Department of Biochemistry and Molecular Biology School of Basic Medicine, Tongji Medical College, Huazhong University of Science and Technology Wuhan China; ^5^ Department of Clinical Laboratory The Central Hospital of Wuhan Wuhan China; ^6^ Department of Pediatrics Tongji Hospital, Tongji Medical College, Huazhong University of Science and Technology Wuhan China; ^7^ Britton Chance Center for Biomedical Photonics‐MoE Key Laboratory for Biomedical Photonics, Advanced Biomedical Imaging Facility, Wuhan National Laboratory for Optoelectronics, Huazhong University of Science and Technology Wuhan China; ^8^ Shenzhen Huazhong University of Science and Technology Research Institute Shenzhen China

**Keywords:** acetylation, Alzheimer's disease, microglia, NLRP3 inflammasome, Tauopathies

## Abstract

**Background:**

Alzheimer's disease (AD) and related Tauopathies are characterised by the pathologically hyperphosphorylated and aggregated microtubule‐associated protein Tau, which is accompanied by neuroinflammation mediated by activated microglia. However, the role of Tau pathology in microglia activation or their causal relationship remains largely elusive.

**Methods:**

The levels of nucleotide‐binding oligomerisation domain (NOD)‐like receptor pyrin domain containing 3 (NLRP3) acetylation and inflammasome activation in multiple cell models with Tau proteins treatment, transgenic mice with Tauopathy, and AD patients were measured by Western blotting and enzyme‐linked immunosorbent assay. In addition, the acetyltransferase activity of Tau and NLRP3 acetylation sites were confirmed using the test‐tube acetylation assay, co‐immunoprecipitation, immunofluorescence (IF) staining, mass spectrometry and molecular docking. The Tau‐overexpressing mouse model was established by overexpression of human Tau proteins in mouse hippocampal CA1 neurons through the adeno‐associated virus injection. The cognitive functions of Tau‐overexpressing mice were assessed in various behavioural tests, and microglia activation was analysed by Iba‐1 IF staining and [18F]‐DPA‐714 positron emission tomography/computed tomography imaging. A peptide that blocks the interaction between Tau and NLRP3 was synthesised to determine the in vitro and in vivo effects of Tau–NLRP3 interaction blockade on NLRP3 acetylation, inflammasome activation, microglia activation and cognitive function.

**Results:**

Excessively elevated NLRP3 acetylation and inflammasome activation were observed in 3xTg‐AD mice, microtubule‐associated protein Tau P301S (PS19) mice and AD patients. It was further confirmed that mimics of ‘early’ phosphorylated‐Tau proteins which increase at the initial stage of diseases with Tauopathy, including TauT181E, TauS199E, TauT217E and TauS262E, significantly promoted Tau–K18 domain acetyltransferase activity‐dependent NLRP3 acetylation and inflammasome activation in HEK293T and BV‐2 microglial cells. In addition, Tau protein could directly acetylate NLRP3 at the K21, K22 and K24 sites at its PYD domain and thereby induce inflammasome activation in vitro. Overexpression of human Tau proteins in mouse hippocampal CA1 neurons resulted in impaired cognitive function, Tau transmission to microglia and microgliosis with NLRP3 acetylation and inflammasome activation. As a targeted intervention, competitive binding of a designed Tau–NLRP3‐binding blocking (TNB) peptide to block the interaction of Tau protein with NLRP3 inhibited the NLRP3 acetylation and downstream inflammasome activation in microglia, thereby alleviating microglia activation and cognitive impairment in mice.

**Conclusions:**

In conclusion, our findings provide evidence for a novel role of Tau in the regulation of microglia activation through acetylating NLRP3, which has potential implications for early intervention and personalised treatment of AD and related Tauopathies.

## Graphical abstract

• Tau directly acetylates NLRP3 at K21, K22 and K24 site at its PYD domain and thereby induce inflammasome activation in vitro.

• Tau overexpression in mouse hippocampal CA1 neurons induces cognitive impairment, Tau transmission to microglia and microgliosis with NLRP3 acetylation and inflammasome activation.

• Competitive binding of a designed TNB peptide to block the interaction of Tau with NLRP3 inhibits the NLRP3 acetylation and downstream inflammasome activation in microglia, thereby alleviating cognitive impairment in mice.

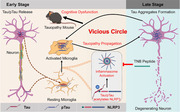

## 1 INTRODUCTION

The microtubule‐associated protein Tau (MAPT) is highly expressed in neuronal axons with the main function of promoting the assembly and stabilisation of microtubules.^1^ Abnormal hyperphosphorylation and aggregation of Tau is usually considered as one of the important hallmarks shared by neurodegenerative Tauopathies including frontotemporal dementia and Parkinsonism linked to chromosome 17 (FTDP‐17), progressive supranuclear palsy, corticobasal degeneration, Pick's disease and Alzheimer's disease (AD).^2^ The hyperphosphorylation of Tau induces its detachment from microtubules and self‐aggregation, triggering the formation of intra‐neuronal toxic Tau aggregates and neurofibrillary tangles (NFTs). These pathological Tau proteins in turn act as prion‐like seeds that are propagated and spread intercellularly, thereby mediating neurodegeneration.^3^, ^4^, ^5^

In the central nervous system (CNS), the extensive interaction between neurons and glial cells (astrocytes, microglia and oligodendrocytes) plays a crucial role in orchestrating physiological brain functions and driving the pathogenesis of multiple Tauopathies.^6^, ^7^, ^8^, ^9^, ^10^ Microglia, the predominant resident macrophages in CNS, are highly plastic and multifunctional cells that continuously monitor the health of neuronal networks by clearing pathogens and dying neurons, and pruning excess neuronal synapses.^11^ β‐Amyloid deposition (Aβ, another major hallmark of AD) can lead to microglial activation, which in turn further aggravates Aβ pathology.^12^, ^13^, ^14^ Interestingly, pathologically phosphorylated Tau (pTau) or the aggregated form of Tau is found to co‐exist with reactive microglia spatially in regions of the brain that are susceptible to Tauopathies.^15^, ^16^, ^17^ Meanwhile, Tauopathies have also been widely demonstrated to directly activate microglia and disturb its homeostasis.^18^, ^19^, ^20^, ^21^ Notably, studies on the brain tissues of patients with Tauopathy have confirmed that Tau pathology is independent of Aβ pathology to trigger microglial activation and neuroinflammation.^16^, ^22^, ^23^ In addition, the activation of microglia promotes further exacerbation and dissemination of Tauopathy, forming a vicious cycle.^24^, ^25^, ^26^ However, the mechanisms underpinning microglia activation in Tau pathology are still unclear. A recent study reported that microglia activation precedes the formation of mature Tau NFTs in the brain of PS19 (P301S) model mice, implying that microglial activation is an early event in Tauopathy.^19^ This is also supported by the findings in brain tissues from patients with Tauopathy.^27^ Therefore, we speculate that Tau/pTau proteins which increase dramatically before extensive Tau aggregation or in the initial stage of the disease^28^, ^29^, ^30^, ^31^ are able to activate microglia via some unknown pathway.

The nucleotide‐binding oligomerisation domain (NOD)‐like receptor pyrin domain containing 3 (NLRP3) inflammasome is an intracellular multimolecular complex consisting of NLRP3, the adaptor molecule apoptosis‐associated speck‐like protein containing a Caspase recruitment domain (ASC) and the pro‐cysteinyl aspartate‐specific proteinase‐1 (pro‐Caspase‐1).^32^ NLRP3 inflammasome has been implicated in several neurodegenerative diseases. It can sense the aggregated pathological proteins including Aβ, α‐synuclein and pTau, and orchestrate the cleavage and secretion of interleukin (IL)‐1β and IL‐18, thereby generating a potent inflammatory response in microglia.^33^, ^34^, ^35^ There is mounting evidence indicating that inflammasome activation in microglia is pivotal for the pathogenesis of Tauopathies.^36^, ^37^ However, the mechanism by which Tau drives NLRP3 inflammasome activation is unknown. Canonical full activation of the NLRP3 inflammasome is regulated by a two‐step process consisting of a priming step at the transcriptional and posttranslational levels, and an activation/assembly step.^38^ Several types of posttranslational modifications (PTMs) such as phosphorylation/dephosphorylation, ubiquitination/deubiquitination, acetylation/deacetylation, SUMOylation and nitrosylation, and protein–protein interactions of NLRP3 inflammasome components license/limit cells for full activation of inflammasome assembly.^39^, ^40^ A recent study showed that suppression of NAD‐dependent deacetylase Sirtuin‐2 mediated acetylation of NLRP3 and promoted NLRP3 inflammasome activation, thus contributing to ageing‐associated inflammation and insulin resistance.^41^ Moreover, nigericin can trigger acetyltransferase KAT5‐mediated NLRP3 acetylation to induce NLRP3 self‐aggregation and full activation of the inflammasome.^42^ Coincidentally, Tau was identified as a bona fide acetyltransferase with sequence and functional similarities to the MYST family of histone lysine acetyltransferases (HATs). It is capable of acetylating itself or other substrates including glycogen synthase kinase‐3β (GSK‐3β), KEAP1 and β‐catenin.^43^, ^44^, ^45^, ^46^ Interestingly, acetyltransferase activity varies among different Tau proteins. For instance, mutant TauS199E, which mimics Tau phosphorylation at Ser199, possesses higher acetyltransferase activity in promoting GSK‐3β and β‐catenin acetylation than wild‐type (WT) Tau441.^44^, ^46^ Based on these findings, we propose that specific type(s) of Tau/pTau may directly acetylate NLRP3 to promote microglial inflammasome activation in Tauopathies.

Herein, we found that NLRP3 acetylation and inflammasome activation were excessively elevated in 3xTg‐AD mice, MAPT P301S (PS19) mice and patients with AD. We further identified that Tau and pTau proteins, which increase at the early stage of Tauopathies significantly promoted NLRP3 acetylation and inflammasome activation in cells, and this effect was dependent on the Tau–K18 functional domain which harbour the acetyltransferase activity. In addition, multiple experiments through methodologies such as immunofluorescence (IF) staining, co‐immunoprecipitation (Co‐IP), in test‐tube acetylation assay and molecular docking confirmed that Tau could directly acetylate NLRP3 at K21, K22 and K24 site at its PYD domain and thereby induce inflammasome activation. Overexpression of human Tau proteins in hippocampal CA1 neurons dramatically impaired the cognitive function of mice, exacerbated Tau spatial transmission to microglia, induced microgliosis with NLRP3 acetylation and inflammasome activation. Lastly, competitive binding of Tau–NLRP3‐binding blocking (TNB) peptide to Tau protein inhibited its acetylation effect on NLRP3 and downstream inflammasome activation in microglia, thereby alleviating cognitive impairment in model mice.

## 2 MATERIALS AND METHODS

### 2.1 Antibodies and reagents

The primary antibodies used in this study are all listed in Table S1. The mouse immunoglobulin G (IgG) and rabbit IgG were purchased from Sigma. The fluorescein and horseradish peroxidase (HRP) labelled secondary antibodies were obtained from Li‐Cor Biosciences and Proteintech Group, respectively. The Alexa Fluor 647‐, 594‐ and 488‐conjugated secondary antibodies were from Thermo Fisher Scientific. The enhanced chemiluminescent substrate was purchased from Biosharp. Neofect DNA transfection reagent was from Neofect Biological Technology Co. Ltd. TPOP146, lipopolysaccharides (LPS) and nigericin were purchased from MedChemExpress. L‐Moses hydrochloride (L‐45) was from GlpBio. The protein marker, dulbecco's modified eagle medium (DMEM), high glucose, DMEM/F12, foetal bovine serum (FBS) and Lipofectamine 3000 were from Invitrogen and Gibco (Thermo Fisher Scientific). Protein A/G agarose was from Smart Lifesciences. All other chemical reagents were standard commercial products with analytical reagent grade.

### 2.2 Human brains and animals

Human brain samples were procured from the China Brain Bank (Zhejiang University School of Medicine). More detailed information is provided in Figure S1C. C57BL/6 male mice (WT) were obtained from the Experimental Animal Center of Tongji Medical College, Huazhong University of Science and Technology. Fifty‐six male C57BL/6 mice aged 12 weeks were randomly divided into four groups for different Tau protein overexpression using adeno‐associated virus (AAV) injection: vehicle (empty vector AAV, *n* = 14), Tau441 (*n* = 15), TauS262E (*n* = 15) and TauK18– (*n* = 12). Three months after virus injection, the behaviours of the mice were tested, which lasted for 2 weeks, followed by positron emission tomography/computed tomography (PET/CT) imaging. Finally, the mice were sacrificed for subsequent molecular biochemical assays and RNA‐Seq of the brain tissues (Figure 4A). In addition, 16 male C57BL/6 mice aged 12 weeks were randomly divided into two groups for subsequent TNB peptide intervention experiment: Tau441 (AAV‐Tau441, *n* = 8) and Tau441 + TNB (*n* = 8). The detailed TNB peptide injection method and other tests can be found in Section 2.13 and Figure 8A. Tau P301S (PS19, strain #: 008169) transgenic mice with age of 3 or 6 months were from Jackson Lab. These mice harbour the T34 isoform and four microtubule‐binding repeats (1N4R) of the Tau protein with P301S mutation under the regulatory control of the murine prion promoter. 3xTg‐AD mice (9‐month old) carrying human mutated APP, PS1 and Tau genes were also from Jackson Lab. During the experiments, all animals were maintained under appropriate temperature (22 ± 1°C), humidity (55 ± 15%) and a 12 h light/12 h dark cycle. All animal experiments were approved by the Animal Care and Use Committee of Huazhong University of Science and Technology and performed in compliance with the NIH Guide for the Care and Use of Laboratory Animals.

### 2.3 Cell culture and plasmid transfection

The plasmid pFLAG‐Tau‐2N4R (Tau441), encoding human Tau, was from our laboratory. The plasmid TauK18– (or TauK18△) lacking microtubule‐binding repeats domain (243–372) was generated by PCR and cloned in a myc‐tagged pcDNA3.0 vector/EGFP N1 vector in EcoRI and BamHI restriction sites. The plasmids GV362‐EGFP‐FLAG‐mouse NLRP3, ‐mouse ASC and ‐mouse Casp1 (fusion with Flag, CMV‐MCS‐3FLAG‐IRES‐EGFP‐SV40‐Neomycin) were provided by Genechem. We also constructed the plasmid pcDNA3.0‐EGFP‐mouse NLRP3 (fusion with EGFP). Mutations of NLRP3 lysine‐21, lysine‐22, lysine‐24 into arginine (K21R, K22R and K24R) and Tau threonine‐181, serine‐199, threonine‐217 and serine‐262 into glutamic acid (T181E, S199E, T217E and S262E) were carried out using the Mut Express II Fast Mutagenesis Kit by following the manufacturer's instructions (Vazyme Biotech). The plasmid SIRT2 was obtained from Addgene (Plasmid #13813).

HEK‐293T, N2a, SH‐SY5Y and BV‐2 microglial cells were cultured in DMEM high glucose medium or DMEM/F12 medium. All cell lines were supplemented with 10% FBS and 1% penicillin/streptomycin, and maintained at 37°C in a humidified atmosphere with 5% CO_2_ and 95% air. The cells were seeded into six‐well plates or 96‐well plates. When the confluence reached to 70%–80%, we added the fresh medium into the cells and then transfected them with relevant plasmids for 24–48 h by Neofect DNA transfection reagent/Lipofectamine 3000 according to the manufacturer's protocols. In addition, primary astrocytes and primary neurons were successfully obtained and cultured according to the previous studies.^47^, ^48^

### 2.4 Cell Counting Kit‐8 cytotoxicity assay

Briefly, HEK‐293T cells were seeded in a 96‐well plate and cultured overnight (80% confluence, 100 µL). After different Tau plasmids transfection (Tau441, TauT181E, TauS199E, TauT217E, TauS262E and TauK18–) for 48 h, the cell viability was assessed by the cell counting kit‐8 (CCK8) assay respectively. CCK8 assay was also used to evaluate the effect of the TNB on the cells including BV‐2, N2a, SH‐SY5Y, primary astrocytes and primary neurons. The peptide TNB/FITC‐TNB with purity of 95.93% (2574.89 Da, LEDLEDVDLKKFKMHLEDYPP) was chemically synthesised by Bioyeargene Biosciences using conventional Fmoc solid‐phase synthetic strategy. The cells were plated and grown to 80% confluence prior to treatment with the peptide with different dosages (0, 25, 50, 100, 200 and 400 µM). After 48 h, the cells were exposed to 10 µL CCK8 reagent for another 4 h. The absorbance at 450 nm was determined using a microplate reader (BioTek SynergyH1). The data from three parallel wells were calculated.

### 2.5 Enzyme‐linked immunosorbent assay

IL‐1β levels in supernatant of BV‐2 cells after corresponding treatments were quantitatively measured through an enzyme‐linked immunosorbent assay (ELISA) using the mouse IL‐1β ELISA kit (Beijing 4A Biotech Co., Ltd.). All detection steps and calculation methods were carried out according to the instructions of the kit.

### 2.6 In vitro assay of NLRP3 inflammasome activation

Based on the previous relevant methods,^49^, ^50^ we reconstituted the mouse inflammasome system in HEK‐293T cells without endogenous NLRP3, ASC and Caspase‐1, aiming to detect Tau‐induced NLRP3 inflammasome activation. This biochemical assay separated ‘priming’ step (S1) from NLRP3 ‘activation’ step (S2) (Figure 2A). In brief, we set up HEK‐293T cells transiently expressing either NLRP3 (293T NLRP3, ‘activator’ or ‘donor’) or ASC and Caspase‐1 (293T ASC‐Casp1, ‘recipient’). It was worth mentioning that excess ASC promotes self‐aggregation and induces NLRP3‐independent Caspase‐1 activation. Thus, the amount and proportion of the plasmids, especially ASC, should be strictly controlled as the following: NLRP3 (2000 ng/well of six‐well plate), ASC (500 ng/well) and Casp1 (2000 ng/well). After NLRP3‐overexpressing cells were treated with the stimulus (S1) such as nigericin (Nig) or overexpressed the Tau proteins (Tau441, TauT181E, TauS199E, TauT217E, TauS262E and TauK18–), the cells were collected in lysis buffer consisting of 10 mM KCl, 1.5 mM MgCl_2_, 10 mM Tris–HCl (pH 7.5), .2% NP‐40 and protease inhibitor cocktail and centrifuged at 1200 *g* for 5 min. The cell extracts (20 µg) were then mixed with 10^6^ recipient cells (S2) semi‐permeabilised with 60 ng perfringolysin O (PFO, Cusabio), a bacterial toxin that forms pores in the plasma membrane. Additional lysis buffer was added to reach a final volume of 20 µL. After incubation at 30°C for 80 min, the reaction mixture was incubated with .5% NP‐40 on ice for 15 min before centrifugation at 16 000 *g* for 15 min. We took part of cell supernatant from S1 or S2 for immunoprecipitation. The remaining supernatant was boiled in sodium dodecyl sulfate (SDS) loading buffer and used for immunoblotting.

### 2.7 Western blotting and immunoprecipitation/co‐immunoprecipitation

The cells and brain tissues were lysed and homogenised with radio immunoprecipitation assay (RIPA) lysis buffer (Beyotime Biotechnology) containing phenylmethanesulfonyl fluoride (PMSF), 1:100 and proteinase inhibitor cocktail (1:100, Yeasen Biotech) before centrifugation for 10 min at 16 000 *g* at 4°C. The obtained supernatant was added to 4× loading buffer (50 mM Tris–HCl, pH 7.6, 2% SDS, 10% glycerol, 10 mM dithiothreitol [DTT]) at the ratio of 3:1, then boiled at 100°C for 10 min followed by sonication and determination of protein concentration by BCA kit (Thermo Fisher Scientific). In order to visualise the protein samples during loading, we added .2% bromphenol blue into them. After electrophoresis in 10% SDS–polyacrylamide gel electrophoresis (PAGE), the separated proteins were transferred onto nitrocellulose membranes (Amersham Biosciences). The membranes were then blocked with 5% nonfat milk dissolved in tris buffered saline (TBS), 50 mM Tris–HCl, pH 7.6, 150 mM NaCl) for 1 h and incubated with primary antibody at 4°C overnight. The membranes were washed by TBST buffer (TBS + .1% Tween‐20) three times for 5 min each and incubated with 1:10 000 anti‐mouse or anti‐rabbit secondary antibody for 1 h at room temperature. The enhanced chemiluminescence kit was then employed to detect signals. The visualised images were collected with Odyssey Infrared Imaging System (Li‐Cor Biosciences) or Chemiscope 6000 (Clinx). For quantification of protein levels, appropriate film exposures were scanned and the density of bands was determined by ImageJ software and normalised to band intensity of β‐actin or GAPDH.

To determine NLRP3 acetylation level and analyse protein–protein interactions, we performed IP/Co‐IP using cell lysates with a diluted concentration of 1 µg/µL. The lysates were incubated with specified primary antibodies and protein A/G agarose beads at 4°C overnight. The beads were collected and washed three times with RIPA buffer/phosphate‐buffered saline (PBS), then resuspended with 1× loading buffer and boiled for 10 min. Finally, the supernatant was collected and analysed by Western blotting.

### 2.8 In test‐tube acetylation assay

To confirm whether Tau protein can acetylate NLRP3, purified human WT full‐length Tau protein (Tau441) and the NLRP3‐protein A/G agarose beads complexes were incubated in test tube (Figure 3D). Purification of Tau441 was accomplished by HUABIO. Briefly, the cDNA of Tau441 was cloned in a His‐tagged PET‐28a (+) vector and amplified in ROSETTA 2(DE3)PLYSS competent cells (1 mM isopropyl β‐D‐thiogalactopyranoside for 4 h induction) before affinity purification by using Ni‐NTA resin. The molecular weight and purity of recombinant Tau are 75 kDa and >85% as estimated by SDS–PAGE gel, respectively. To obtain NLRP3 protein, we transfected the plasmid GV362‐EGFP‐FLAG‐NLRP3 (or the mutant NLRP3) into HEK‐293T cells in a 10 cm diameter dish for 48 h. The NLRP3 protein in cells was completely recruited to agarose beads by IP with anti‐Flag primary antibody. For the in test‐tube acetylation assay, 50 nM each of the purified Tau protein, one‐third of the NLRP3‐protein A/G agarose beads complexes and 1 mM acetyl coenzyme A (acetyl CoA, Sigma) were incubated at 37°C for 2 h with constant shaking in the reaction buffer (50 mM HEPES [pH 8.0], 10 mM sodium butyrate, 1 mM DTT and 10% glycerol).^46^ The acetylation level of NLRP3 was analysed by Western blotting using anti‐acetylated lysine antibody. Using this system, we also detected the in vitro binding between purified Tau and NLRP3 in this assay.

### 2.9 Immunofluorescence and confocal microscopy imaging

The mice under isoflurane anesthesia were perfused intracardially with normal saline and 4% paraformaldehyde (PFA) to pre‐fix the brain tissues. Immediately, the mice were euthanised and the brains were quickly removed into fresh 4% PFA for a further 24 h fixation at 4°C. After washing with PBS, the brains were immersed into 30% sucrose–PBS for thorough dehydration. Then, the brains were embedded in opti‐mum cutting temperature (OCT compound), frozen and coronally cut into 30 µm sections using a cryotome (CM1950, Leica). Slices were permeabilised with .5% Triton–PBS and blocked with 3% bovine serum albumin (BSA) for 30 min, respectively. After staining with indicated antibodies (overnight for primary antibodies staining and 1 h for Alexa Fluor 647‐, 594‐ or 488‐conjugated secondary antibodies staining), they were incubated with 4',6‐diamidino‐2‐phenylindole (DAPI) for 8 min at room temperature. For cultured cells, the cells were washed three times with PBS for 5 min after indicated treatment, and then fixed with 4% PFA for 30 min at 4°C. The remaining steps were the same as described in the brain slice samples above. Eventually, the samples were transferred to microscope slides for observation and images acquisition under a LSM800 fluorescence microscope (Zeiss) or a VS120 virtual slide microscope (Olympus). All quantitative and morphological analyses were fulfilled by using ImageJ software.^51^

### 2.10 Mass spectrometry analysis

After Tau‐induced acetylation reaction (Figure 3D), the NLRP3–protein A/G agarose beads complexes were washed with RIPA buffer/PBS three times. Then, we added 40 µL 1× loading buffer into the complexes, boiled the sample for 10 min and performed SDS–PAGE and Coomassie blue staining. Protein samples in gel (about 1 cm × 1 cm in size) were collected and sent to SpecAlly Life Technology Co., Ltd. for liquid chromatography–mass spectrometry (MS) analysis.

### 2.11 Molecular docking

Molecular docking was performed in the ClusPro server (https://cluspro.org)^52^ using existing NLRP3 PYD monomer structure (PDB: 2NAQ) and Tau filament structure (PDB: 7NRS). The docking results were modified in Coot before going through the PDBePISA server. The potential docking solutions with low energy were chosen since they all displayed Tau–NLRP3 interaction and relevant sites. Structure images were generated using the PyMOL Molecular Graphics System.

### 2.12 Stereotaxic injection

C57BL/6 mice under deep anesthesia were injected with the AAV viruses (AAV9 serotype) by using a stereotaxic instrument (Kopf). To achieve transfection of neurons in vivo, we selected the neuron‐specific promoter hSyn. The AAV viruses including pAAV9‐hSyn‐Tau441‐mCherry‐3FLAG (Tau441), AAV9‐hSyn‐TauS262E‐mCherry‐3FLAG (TauS262E), AAV9‐hSyn‐TauK18△‐mCherry‐3FLAG (TauK18–) and control vector (vehicle) were constructed and packaged by Genechem. Left hippocampal CA1 region stereotaxic injections (−1.9 mm anterior/posterior [A/P], −.7 mm medial/lateral to bregma [M/L] and −1.5 mm dorsal/ventral [D/V] to the dura surface) of 2 µL high‐titre AAV (∼10^13^ v.g./mL) were performed with a Hamilton syringe at a rate of .2 µL/min. Three months later, the mice were tested for the behaviours and PET imaging, and then sacrificed for further detections (Figure 4A).

### 2.13 In vivo peptide administration

Two weeks after pAAV9‐hSyn‐Tau441‐mCherry‐3FLAG injection into the left hippocampal CA1, C57BL/6 mice were repeatedly administered with the TNB peptide of 5 mM via lateral ventricle (−.2 mm A/P, −.9 mm M/L, −2.3 mm D/V)‐implanted guiding cannulas (RWD) once every 3 days for 6 weeks. During dosing, mice were restricted by a stereotaxic apparatus and kept awake. One month after the last peptide administration, the mice were tested for the behaviours and sacrificed to further detections (Figure 8A). To confirm the effective delivery of the FITC–TNB peptide, we also injected the peptide into lateral ventricle of C57BL/6 mice for 1 h and euthanised them immediately to observe the location of TNB in brain through confocal microscopy imaging.^53^

### 2.14 Behaviour tests

In open field test (OFT), the mice were put into a square‐shaped 50 cm × 50 cm plastic apparatus surrounded by a 40 cm wall, and allowed to freely explore for 5 min. A 25 × 25 (or 30 × 30) cm^2^ square area at the centre of the apparatus was defined as the centre zone. The mice were first placed into the centre zone and their behaviour record was started simultaneously. Data were gathered and analysed with a video‐tracking system (Chengdu Techman Software Co., Ltd.). After each trial, the open field was cleaned with a paper towel dampened with 75% ethanol to remove odour cues. For analysis, the ratio of time spent in the centre zone to the total time was quantified.

In novel object recognition (NOR) test, for adapting the environment, the mice were put in the same plastic apparatus as in OFT for 5 min without any other object 24 h before the experiment. On the next day, the mice re‐entered the apparatus from the same starting point and were allowed to freely explore the symmetrically placed objects A and B (different shape and colour) for 5 min. To measure long‐term memory, after 24 h, the object B was replaced by object C with different shape and colour from A and B, and the mice were reintroduced into the apparatus to explore for 5 min (test phase). Between mice trials, the equipment was wiped with ethanol to remove odour cues. The exploration behaviour of mice on the two objects (A/B and A/C) was recorded using a video‐tracking system (Chengdu Techman Software Co., Ltd.). The novel object discrimination index was calculated as the time spent in exploring the novel object divided by the time spent on both objects, which was quantified in both the training phase and test phase.

In fear conditioning test (FCT), the mice were placed into the conditioning chamber (33 cm × 33 cm × 33 cm) equipped with white board walls, a transparent front door, a grid floor and a speaker (Chengdu Techman Software Co., Ltd.). On day 1, the mice were allowed to conduct a free exploration for 3 min before delivery of a tone conditioned stimulus (CS, 20 s, 80 dB, 300 Hz) through the speaker paired with a foot shock unconditioned stimulus (US, 3 s, .8 mA) at the end of the tone through the grid floor. A total of three CS–US pairs with a 60 s intertrial interval (ITI) were exerted to each animal in the training stage. The mouse was removed from the chamber 1 min after the last foot shock and placed back into the home cage. The contextual fear conditioning stage started 24 h after the training phase. During the test, the mouse was put back into the same conditioning chamber without sound stimulation for 5 min, and the time of its freezing responding to the same context was recorded. On the third day, the mouse was placed back into the same chamber with different contextual cues including coloured walls and a smooth plastic floor for 5 min. After 2 min of free exploration, the mouse was exposed to the exact same 3‐CS tones with 20 s ITI as in the training stage without the foot shock, and its freezing response to the tone in the altered context was recorded.

The Morris water maze (MWM) was performed as previously described to detect spatial learning and memory.^54^, ^55^ The apparatus used in MWM was a circular tank (120 cm in diameter, 60 cm in height) filled with nontoxic white dye‐tinted water (25 ± 2°C). Different posters are plastered on the walls of the tank. The maze was theoretically divided into four equal quadrants. An escape platform with a diameter of 10 cm was placed in the middle of one of the quadrants (target quadrant) and submerged 1 cm below the water surface and kept in the same position during the trial. A video‐tracking camera above the centre of the pool surface monitored the trajectory of the mice, and the video signal was transmitted to a computer in an adjacent room. Each mouse underwent four training trials a day for consecutive 5 days from semi‐random start positions to find the hidden platform within 60 s. If the mouse could find the platform, it would stay on the platform for 20 s. Otherwise, the mouse would be gently directed to the platform and kept there for 20 s. Latency (s) to find the hidden platform was recorded after each trial of each learning session. The hidden platform was removed 48 h after the end of training. The mice were placed into the pool for the probe trial with a duration of 60 s. The target quadrant crossing times was recorded.

### 2.15 PET/CT imaging

As previous procedure,^56^ we labelled Tosylate‐DPA‐714 (CAS: 958233‐17‐5, Huayi Isotopes) with 18F produced by a cyclotron (MINItrace, GE Healthcare) to obtain the radiotracer [18F]‐DPA‐714 (radiochemical purity [RCP] = 99%) using a TRACERlab FX FN synthesis module (GE Healthcare). The RCP was determined by means of HPLC, using an U3000 HPLC system (Thermo Scientific) with a Flow‐Ram radio‐HPLC detector (LabLogic, Sheffield). Simultaneous PET and CT scanning in mouse brain was performed by using a small animal microPET/SPECT/CT multimodal imaging system (InliView‐3000B, Novel Medical, Yongxin Medical Equipment Co., Ltd.). The mice were fasted for 12 h and weighted before the radiotracer injection. Each mouse was intravenously injected with 200 µCi ± 10 µCi of [18F]‐DPA‐714 via tail vein. After an uptake period of 50 min, the mice were quickly anaesthetised with isoflurane inhalation and prone positioned in the PET/CT scanning bed board with a mask covering its mouth to ensure continuous anesthetic gas inhalation throughout the scanning. CT localisation was first conducted to select the head as the scanning range of CT and PET. PET scan was performed for 10 min. Image analysis was performed using PMOD v4.104 (PMOD Technologies). A predefined mouse brain atlas template was used to analyse different brain regions including striatum, cortex, hippocampus, thalamus and cerebellum. Standard uptake value (SUV) of [18F]‐DPA‐714 in the region of interest (ROI) was calculated as: SUV = the radioactivity concentration of ROI (kBq/mL)/injected dose (MBq)/body weight (kg), 1 µCi = .037 MBq.

### 2.16 Statistical analysis

All data were expressed as means ± SEMs of at least three independent assays unless otherwise stated. Statistical analyses were carried out using GraphPad Prism 7.0 (GraphPad Software Inc.). Differences between groups were evaluated by unpaired two‐tailed Student's *t* test or one‐way analysis of variance. Correlations between groups were determined by Pearson's correlation test. *p *< .05 was considered statistically significant: ^****^ *p *< .0001, ^***^ *p *< .001, ^**^ *p *< .01 and ^*^ *p *< .05.

## 3 RESULTS

### 3.1 NLRP3 inflammasome is activated with increased NLRP3 acetylation in Tauopathy transgenic mice and AD patients

**FIGURE 1:**
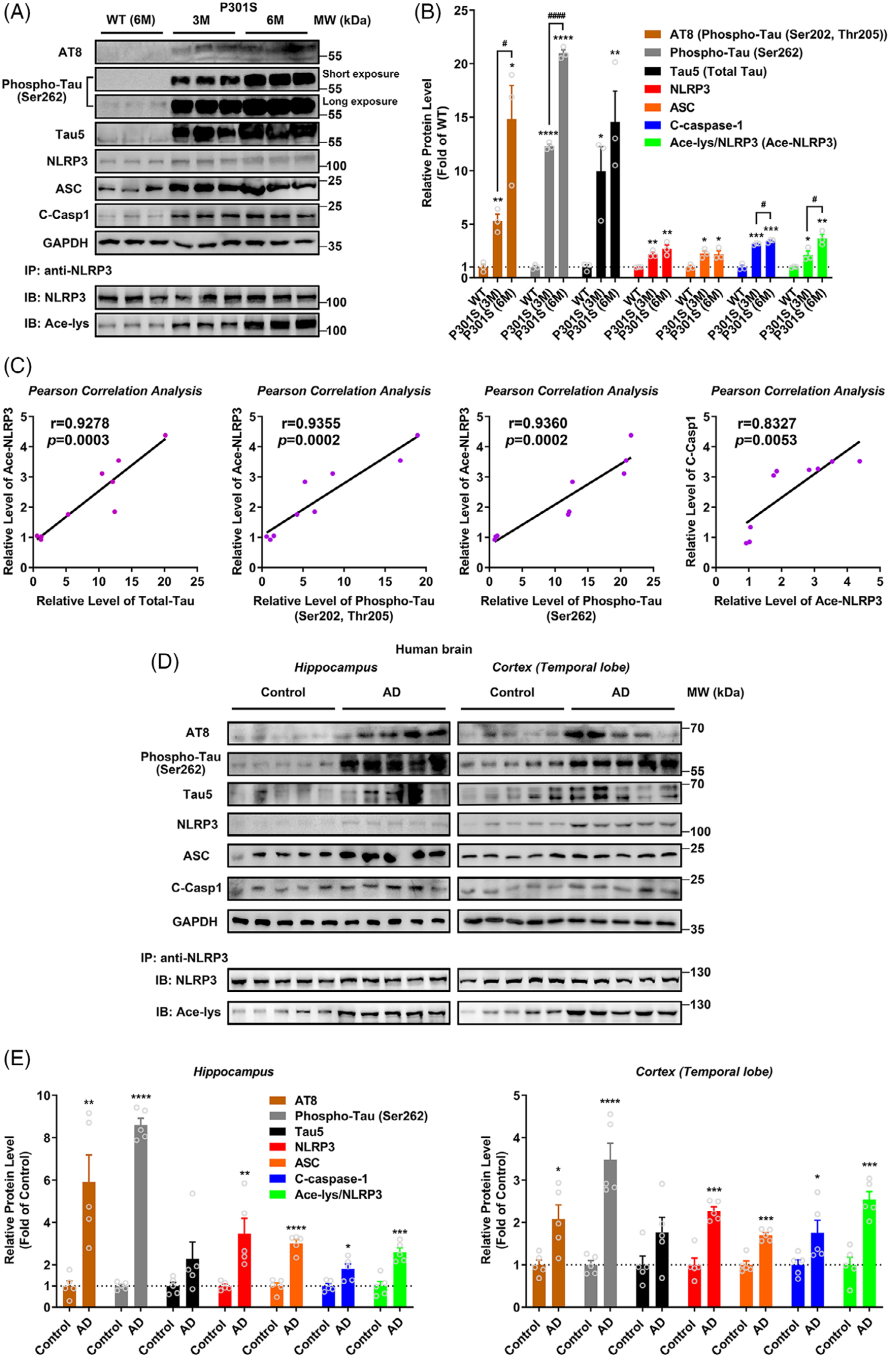
Nucleotide‐binding oligomerisation domain (NOD)‐like receptor pyrin domain containing 3 (NLRP3) inflammasome is activated with increased NLRP3 acetylation in Tauopathy transgenic mice and Alzheimer's disease (AD) patients. (A) Immunoblot of phosphorylated Tau (AT8 and Phospho‐Tau at Ser262), total Tau (Tau5), NLRP3, ASC, cleaved Caspase‐1 (C‐Casp1) and GAPDH as well as acetylated NLRP3 (Ace‐NLRP3, IB: Ace‐lys/IB: NLRP3) in the hippocampus of wild‐type (WT) mice at 6 months of age and P301S mice at 3 and 6 months of age. (B) Quantification of immunoblots in (A). *n* = 3 mice per group. ^*^ *p* < .05, ^**^ *p* < .01, ^***^ *p* <.001, ^****^ *p* < .0001 versus WT group, ^#^ *p* < .05, ^####^ *p* < .0001. (C) Correlation analysis between total Tau and Ace‐NLRP3 (*r* = .9278, *p* = .0003), between phosphorylated Tau AT8 (Phospho‐Tau (Ser202, Thr205)) and Ace‐NLRP3 (*r* = .9355, *p* = .0002), between phosphorylated Tau (Phospho‐Tau (Ser262)) and Ace‐NLRP3 (*r* = .9360, *p* = .0002), and between Ace‐NLRP3 and C‐Casp1 (r = .8327, *p* = .0053) in WT/P301S mice based on the data from (A) and (B). (D) Immunoblots of the proteins same as in (A) in the hippocampus and cortex of AD patients (AD) and controls (control). (E) Quantification of immunoblots in (D). *n* = 5 for Control and AD group. ^*^ *p* < .05, ^**^ *p* < .01, ^***^ *p* < .001, ^****^ *p* < .0001 versus control group.

To identify the potential role of NLRP3 acetylation in inflammasome activation in transgenic mice and patients with Tauopathy, we analysed hippocampal samples from 3xTg‐AD transgenic mice (9‐month old). The elevated Caspase‐1 cleavage and NLRP3 and ASC levels indicated NLRP3 inflammasome activation, and meanwhile NLRP3 acetylation increased in AD mice (Figure S1A,B). Although we observed a great increase of Tau/pTau (AT8 and Phospho‐Tau (Ser262)), it is noteworthy that extracellular Aβ deposits commonly arise in 3xTg‐AD transgenic mice at 6 months or older.^57^ Thus, to further identify the potential relationship between Tau and NLRP3 acetylation, we tested the hippocampal samples from PS19 transgenic mice with age of 3 or 6 months. PS19 mouse line overexpresses MAPT with the P301S mutation and is a representative Tauopathy model. As shown in Figure 1A,B, the levels of NLRP3, ASC, cleaved Caspase‐1 as well as Ace‐NLRP3 (Ace‐lys/NLRP3) in hippocampal samples from 3‐ or 6‐month‐old PS19 mice were significantly increased compared with 6‐month‐old WT mice. More importantly, NLRP3 acetylation and NLRP3 inflammasome activation were also observed in the hippocampal tissues and temporal cortices of AD patients (Figure 1D,E). Pearson correlation analysis based on the above results revealed the strong correlations among Tau/pTau level, NLRP3 acetylation and NLRP3 inflammasome activation: Tau/pTau level was positively correlated with NLRP3 acetylation, and NLRP3 acetylation level was positively correlated with inflammasome activation (Figures 1C and S1D). These data suggest that NLRP3 acetylation is increased with inflammasome activation in Tauopathy transgenic mice and patients with AD, and Tau/pTau level is highly correlated with NLRP3 acetylation and inflammasome activation.

### 3.2 Tau promotes NLRP3 acetylation and inflammasome activation dependent on K18 acetyltransferase activity domain

**FIGURE 2:**
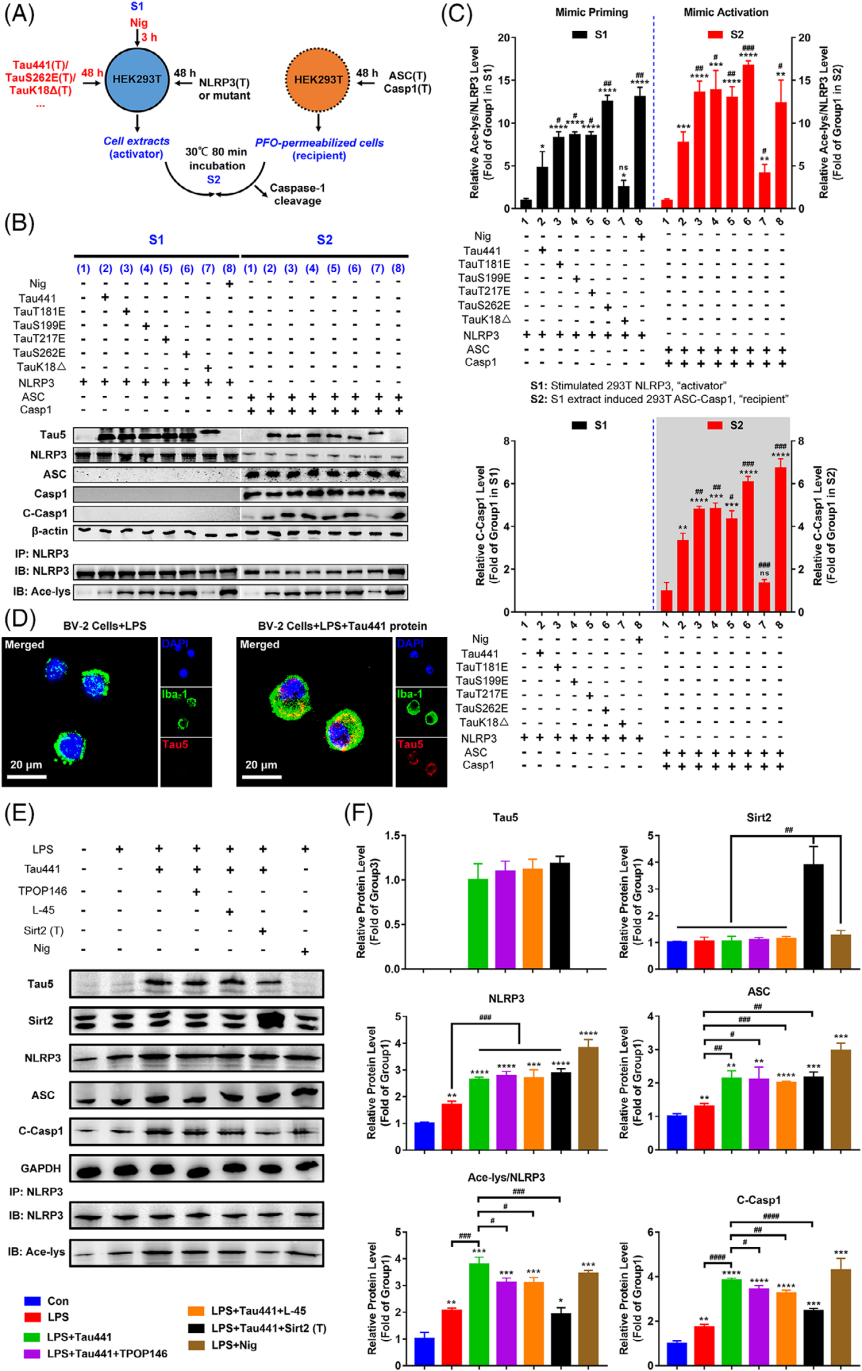
Tau promotes nucleotide‐binding oligomerisation domain (NOD)‐like receptor pyrin domain containing 3 (NLRP3) acetylation and inflammasome activation, which requires K18 acetyltransferase activity‐harbouring domain. (A) Diagram of in vitro assay of NLRP3 inflammasome activation upon different Tau isoform treatment: HEK‐293T donor cells transiently expressing NLRP3 were co‐transfected with different Tau proteins (Tau441, TauT181E, TauS199E, TauT217E, TauS262E and TauK18–) for 48 h or stimulated with nigericin (Nig, 20 µM, positive control) for 3 h (S1). Cell extracts were collected and mixed with perfringolysin O (PFO)‐permeabilised HEK‐293T cells transiently expressing ASC and Caspase‐1 (recipient cells) at 30°C for 80 min. After incubation, the reaction mixture was analysed by immunoblotting for Caspase‐1 cleavage (S2). (B) Representative immunoblot of transfected Tau (Tau5), NLRP3, ASC, Caspase‐1 (Casp1), cleaved Caspase‐1 (C‐Casp1), β‐actin and acetylated NLRP3 (Ace‐NLRP3) in S1 and S2. Group 1: NLRP3 (S1)/ASC + Casp1 (S2); group 2: Tau441 + NLRP3 (S1)/ASC + Casp1 (S2); group 3: TauT181E + NLRP3 (S1)/ASC + Casp1 (S2); group 4: TauS199E + NLRP3 (S1)/ASC + Casp1 (S2); group 5: TauT217E + NLRP3 (S1)/ASC + Casp1 (S2); group 6: TauS262E + NLRP3 (S1)/ASC + Casp1 (S2); group 7: TauK18Δ + NLRP3 (S1)/ASC + Casp1 (S2); group 8: Nig + NLRP3 (S1)/ASC + Casp1 (S2). (C) Quantification of C‐Casp1 and Ace‐NLRP3 in (C). *n* = 3, ns represents no significant difference, ^*^ *p* < .05, ^**^ *p* < .01, ^***^ *p* < .001, ^****^ *p* < .0001 versus group 1, ^#^ *p* < .05, ^##^ *p* < .01, ^###^ *p* < .001 versus group 2. (D) BV‐2 cells pretreated with 1 µg/mL LPS for 4 h (priming) were incubated with or without purified Tau441 protein (5 µM) for 18 h, representative immunofluorescence confocal images showing uptake of exogenous purified Tau441 protein into BV‐2 microglial cells. Scale bar = 20 µm. (E) BV‐2 cells pretreated with 1 µg/mL LPS for 4 h (priming) were incubated with purified Tau441 protein (5 µM) for 18 h or Nig for 3 h as positive control, and NLRP3 acetylation and inflammasome activation were detected. For confirming the key effect of Tau‐induced acetylation in NLRP3 inflammasome activation, cells were simultaneously treated with lysine acetyltransferase inhibitor TPOP146 (134 nM) or L‐45 (126 nM), or overexpressed Sirt2 to reduce the acetylation levels. Representative immunoblot of internalised Tau441 protein (Tau5), Sirt2, NLRP3, ASC, C‐Casp1, GAPDH and Ace‐NLRP3. (F) Quantification of the protein levels in (E). *n* = 3, ^*^ *p* < .05, ^**^ *p* < .01, ^***^ *p* < .001, ^****^ *p* < .0001 versus Con group, ^#^ *p* < .05, ^##^ *p* < .01, ^###^ *p* < .001, ^####^ *p* < .0001 as indicated.

In order to determine the role of Tau proteins in NLRP3 acetylation and inflammasome activation in microglia, according to disease progression and Tau‐associated early diagnostic markers,^31^ we chose to explore the effects of four representative pTau with relatively high abundances in the early stage of disease: pTau‐T181, pTau‐S199, pTau‐T217 and pTau‐S262 through overexpressing their mimics TauT181E, TauS199E, TauT217E and TauS262E in cells. Additionally, as controls, full‐length WT Tau441 and microtubule‐binding repeats domain lacked TauK18‐overexpression plasmids were used. As shown in Figure S2A, after 4 h pretreatment with 1 µg/mL LPS, the BV‐2 microglia overexpressed Tau protein for 48 h (the transfection efficiency of GFP‐Tau is relatively low, at 18.4%), or was incubated with the supernatant/lysate from HEK‐293T cells with Tau protein overexpression for 18 h or nigericin (Nig, inflammasome activation positive control, 20 µM) for 3 h. Tau overexpression in HEK‐293T cells did not reduce the viability of cells, and resulted in Tau release into the culture medium (Figure S2B,D). All the three treatments on BV‐2 cells, including incubation with the supernatant (S) or lysate (P) from Tau‐overexpressing HEK‐293T cells, or direct overexpression (T) of WT Tau441 or pTau mimics, induced NLRP3 inflammasome activation, manifested by significantly increased NLRP3, ASC and cleaved Caspase‐1 levels as well as IL‐1β release (Figure S2E–K). By comparison, the supernatant and cell lysate containing TauS262E protein induced the strongest NLRP3 inflammasome activation in BV‐2 cells, followed by TauT181E, TauS199E, TauT217E and then Tau441, while TauK18– had no effect on promoting NLRP3 inflammasome activation compared with LPS treatment alone group (Figure S2E–H). Tau also induced NLRP3 acetylation in BV‐2 cells, and TauS262E again, showed the strongest effect, whereas TauK18– had no impact on NLRP3 acetylation (Figure S2I,J). Similarly, Pearson correlation analysis at the cellular level indicated that NLRP3 acetylation may contribute to inflammasome activation (Figure S2L). To verify the acetylation of NLRP3 and activation of inflammasome induced by Tau and pTau, we next adopted a more accurate method to evaluate the activity of NLRP3 inflammasome based on HEK‐293T cell culture system (Figure 2A). Tau/NLRP3 and ASC/Caspase‐1 were separately expressed in donor and recipient cells. The latter, after PFO permeabilisation, were incubated with extracts from Tau/NLRP3‐overexpressing donor cells (activator), then the cleavage of Caspase‐1 was detected to access the NLRP3 inflammasome activation. As shown in Figure 2B,C, in donor cells, full‐length Tau and pTau‐induced NLRP3 acetylation, with TauS262E showing the most pronounced effect; when recipient cells with ASC/Caspase‐1 expression were incubated with the Tau‐stimulated NLRP3 from donor cells, Caspase‐1 was cleaved, indicating effective NLRP3 inflammasome assembly and activation. These results demonstrated that Tau/pTau‐induced NLRP3 acetylation can potently increase Caspase‐1 cleavage, whereas TauK18– had no effect on NLRP3 acetylation or Caspase‐1 cleavage. By comparing the NLRP3 acetylation level in Tau treated groups in S1 and S2, it could be found that NLRP3 acetylation levels were further increased during the 80‐min incubation, implying that Tau protein continue exerting its effect of acetylating NLRP3 in S2. By contrast, NLRP3 did not further increase its acetylation level in S2 in positive control group since in this incubation system the acetylation inducer and NLRP3 activator nigericin^42^ was not added. Next, acetylation of NLRP3 induced by Tau was further identified by incubation of BV‐2 cells with purified Tau protein. After 4 h pretreatment with 1 µg/mL LPS, BV‐2 cells were incubated with purified Tau441 protein (5 µM, by referring to the previous publications^58^, ^59^) with or without Sirt2 overexpression/acetyltransferase inhibitor TPOP146 or L‐45 treatment for 18 h. As shown, exogenous recombinant Tau441 protein was found in BV‐2 cells (Figure 2D), and induced NLRP3 acetylation and inflammasome activation, which could be prevented by deacetylase Sirt2 and acetyltransferase inhibitor TPOP146/L‐45 (Figure 2E,F). Together, these data suggest that Tau and pTau proteins which increase in early stage of Tauopathy promote NLRP3 acetylation and activate NLRP3 inflammasome.

### 3.3 K21, K22 and K24 at NLRP3‐PYD domain are key sites for Tau‐induced NLRP3 acetylation and inflammasome activation

**FIGURE 3:**
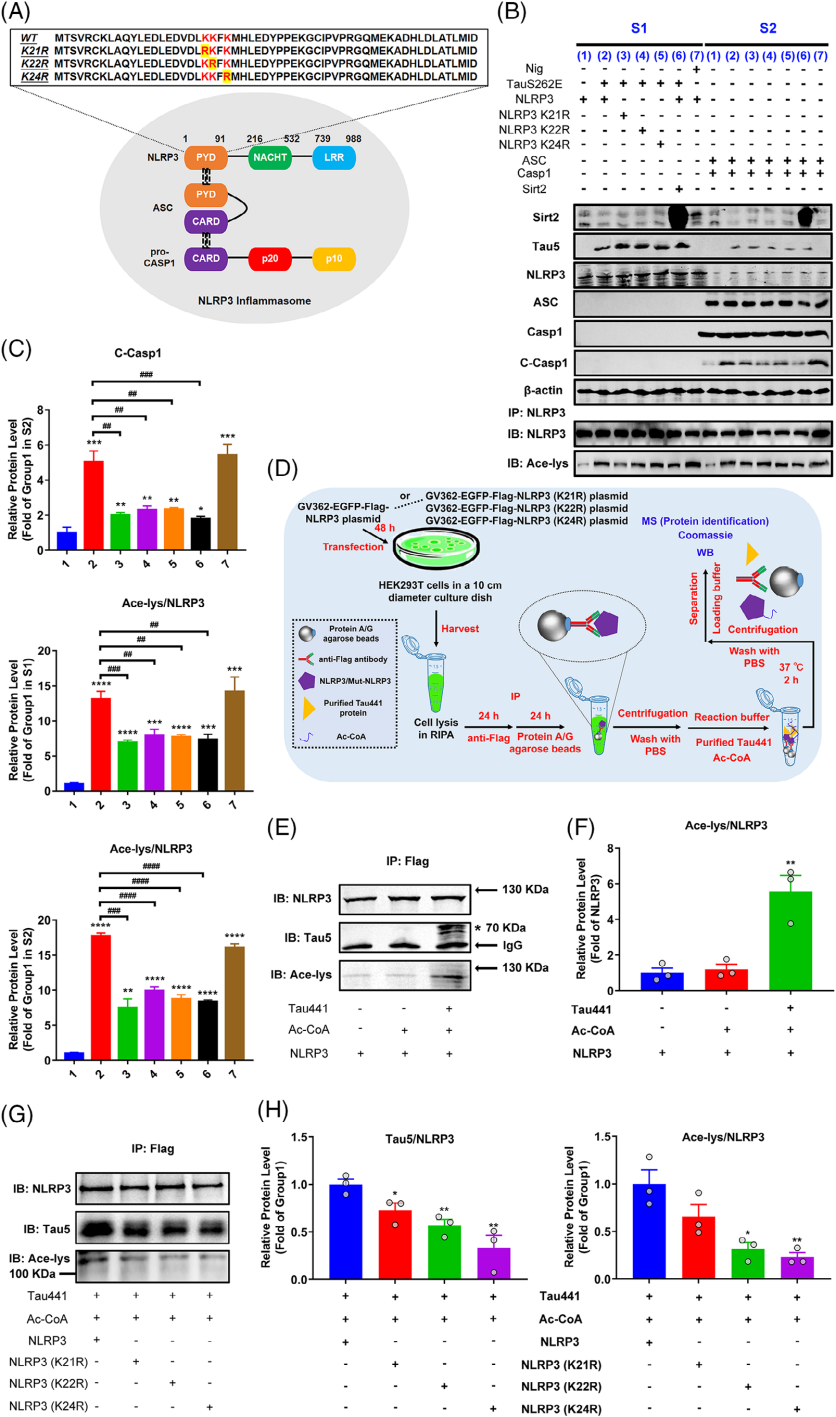
K21, K22 and K24 at nucleotide‐binding oligomerisation domain (NOD)‐like receptor pyrin domain containing 3 (NLRP3)‐PYD domain are key sites for Tau‐induced NLRP3 acetylation and inflammasome activation. (A) Schematic representation of NLRP3 inflammasome and NLRP3–PYD domain including wild type (WT) and mutants (K21R, K22R and K24R). (B) HEK‐293T cells were co‐transfected with WT or mutant NLRP3 (NLRP3 K21R, NLRP3 K22R or NLRP3 K24R) and TauS262E for 48 h, with or without simultaneous overexpression of NLRP3 deacetylase Sirt2, or stimulated with nigericin (Nig, 20 µM, positive control) for 3 h (S1). Cell extracts were collected and mixed with perfringolysin O (PFO)‐permeabilised HEK‐293T cells transiently expressing ASC and Caspase‐1 at 30°C for 80 min. After incubation, the reaction mixture was analysed by immunoblotting for Caspase‐1 cleavage (S2) for evaluation of NLRP3 inflammasome activation. Representative immunoblots of Sirt2, transfected Tau (Tau5), NLRP3, ASC, Caspase‐1 (Casp1), cleaved Caspase‐1 (C‐Casp1), β‐actin and acetylated NLRP3 (Ace‐NLRP3) in S1 and S2. Group 1: NLRP3 (S1)/ASC + Casp1 (S2); group 2: TauS262E + NLRP3 (S1)/ASC + Casp1 (S2); group 3: TauS262E + NLRP3 K21R (S1)/ASC + Casp1 (S2); group 4: TauS262E + NLRP3 K22R (S1)/ASC + Casp1 (S2); group 5: TauS262E + NLRP3 K24R (S1)/ASC + Casp1 (S2); group 6: TauS262E + NLRP3 + Sirt2 (S1)/ASC + Casp1 (S2); group 7: Nig + NLRP3 (S1)/ASC + Casp1 (S2). (C) Quantification of C‐Casp1 in S2 and Ace‐NLRP3 in S1 and S2. *n* = 3, ^*^ *p* < .05, ^**^ *p* < .01, ^***^ *p* < .001, ^****^ *p* < .0001 versus group 1, ^##^ *p* < .01, ^###^ *p* < .001, ^####^ *p* < .0001 versus group 2. (D) Schematic diagram of detection of NLRP3 acetylation induced by purified Tau441 protein. HEK‐293T cells were transfected with GV362‐EGFP‐Flag‐NLRP3 or K21R, K22R, K24R mutant NLRP3 plasmid for 48 h, then NLRP3 was immunoprecipitated by anti‐Flag antibody, and incubated with purified Tau441 protein and acetyl coenzyme A (Ac‐CoA) in test‐tube containing reaction buffer at 37°C for 2 h. (E) The acetylation of NLRP3 and its binding with Tau441 protein were detected by immunoblotting using anti‐NLRP3, anti‐acetylated‐lysine (Ace‐lys) and anti‐Tau5 antibody. The target band is marked with ‘*’. (F) Quantification of the Ace‐NLRP3 level using the ratio of Ace‐lys to NLRP3. *n* = 3, ^**^ *p* < .01 versus NLRP3 group. (G) The ability of Tau441 protein binding with different NLRP3 proteins (WT, K21R, K22R and K24R) and its effect on different NLRP3 acetylation were measured by immunoblotting using anti‐NLRP3, anti‐Ace‐lys and anti‐Tau5 antibody. (H) Quantification of the data from (G). The ratio of Tau5 to NLRP3 reflects the binding ability. *n* = 3, ^*^ *p* < .05, ^**^ *p* < .01 versus group ‘Tau441 + Ac‐CoA + NLRP3’.

It has been reported that NLRP3 K21 and K22 sites are modified by acetylation in macrophages and are targeted by an NAD+‐dependent deacetylase Sirt2 for deacetylation, and NLRP3 acetylation facilitates the NLRP3 inflammasome activation.^41^ In addition, K24 acetylation of NLRP3 regulated by KAT5, a histone acetyltransferase belonging to MYST family, is critical for its full activation.^42^ Coincidentally, Tau was identified as a bona fide acetyltransferase that shares sequence and functional similarities to the MYST family of HATs, and is capable of self‐acetylation.^43^ Therefore, we tested whether these sites were involved in Tau‐induced NLRP3 acetylation by overexpressing mutant plasmids of NLRP3 (K21R, K22R and K24R) and Sirt2 in TauS262E treated HEK‐293T cell model (Figures 2A and 3A). The results showed that the levels of TauS262E‐induced NLRP3 acetylation, as well as NLRP3 inflammasome activation, were significantly decreased in K21R, K22R and K24R mutant‐overexpressing cells (Figure 3B,C). Likewise, Sirt2 overexpression also significantly suppressed Tau‐induced NLRP3 acetylation and inflammasome activation (Figure 3B,C). Altogether, these results indicate that the highly conserved residues K21, K22 and K24 in the NLRP3‐PYD domain are potential sites of Tau‐induced NLRP3 acetylation, which is crucial for the activation of NLRP3 inflammasome.

Although the previous results demonstrated that different Tau proteins exhibited varying effects on promoting NLRP3 acetylation and inflammasome activation, there was no evidence supporting a direct interaction between Tau and NLRP3. To confirm whether Tau directly acts on NLRP3, we first transfected EGFP–NLRP3 and Flag–Tau441 plasmids into HEK‐293T cells for 48 h, and then performed IF staining or Co‐IP (Figure S3A). As shown in Figure S3B,C, Tau visibly co‐localized with and was bound to NLRP3. Next, we incubated purified WT Tau441 protein with agarose beads bound to NLRP3 or mutant NLRP3 proteins (K21R, K22R and K24R NLRP3) in the test tube, and then measured the levels of NLRP3 acetylation and Tau–NLRP3 binding (Figure 3D). We observed the binding of Tau protein to NLRP3 again and confirmed the acetyltransferase activity of Tau441 protein in NLRP3 acetylation, while the mutations of K21R, K22R and K24R markedly impeded the binding and subsequent acetylation (Figure 3E–H), which convincingly illustrates that Tau can directly bind to and acetylate NLRP3, and K21, K22 and K24 at NLRP3–PYD domain are key sites for Tau‐induced NLRP3 acetylation. Coomassie staining combined with MS detection revealed the presence of Tau protein on the agarose beads (Figure S3D,E), which further suggested the direct binding of Tau and NLRP3. Since NLRP3 K21, K22 and K24 mutations dampened the NLRP3 binding to Tau and NLRP3 acetylation induced by Tau, we speculated that the region containing these sites might mediate the interaction with Tau. Therefore, we conducted molecular docking to confirm the interaction region between Tau and NLRP3. As shown in Figure S3F, three potential docking models displayed that K21 and K22 sites of NLRP3 were in close proximity (<3.0 Å) to the Tau S324 and D348 sites, which met our expectation. In addition, MS detection of the Tau‐incubated NLRP3 identified abundant conserved NLRP3 unique peptide LAQYLEDLEDVDLKK containing K21 and K22 sites, reinforcing the possibility of their acetylation modification by Tau (Figure S3E).

### 3.4 Tau overexpression in hippocampus induces cognitive impairment in C57BL/6 mice

**FIGURE 4:**
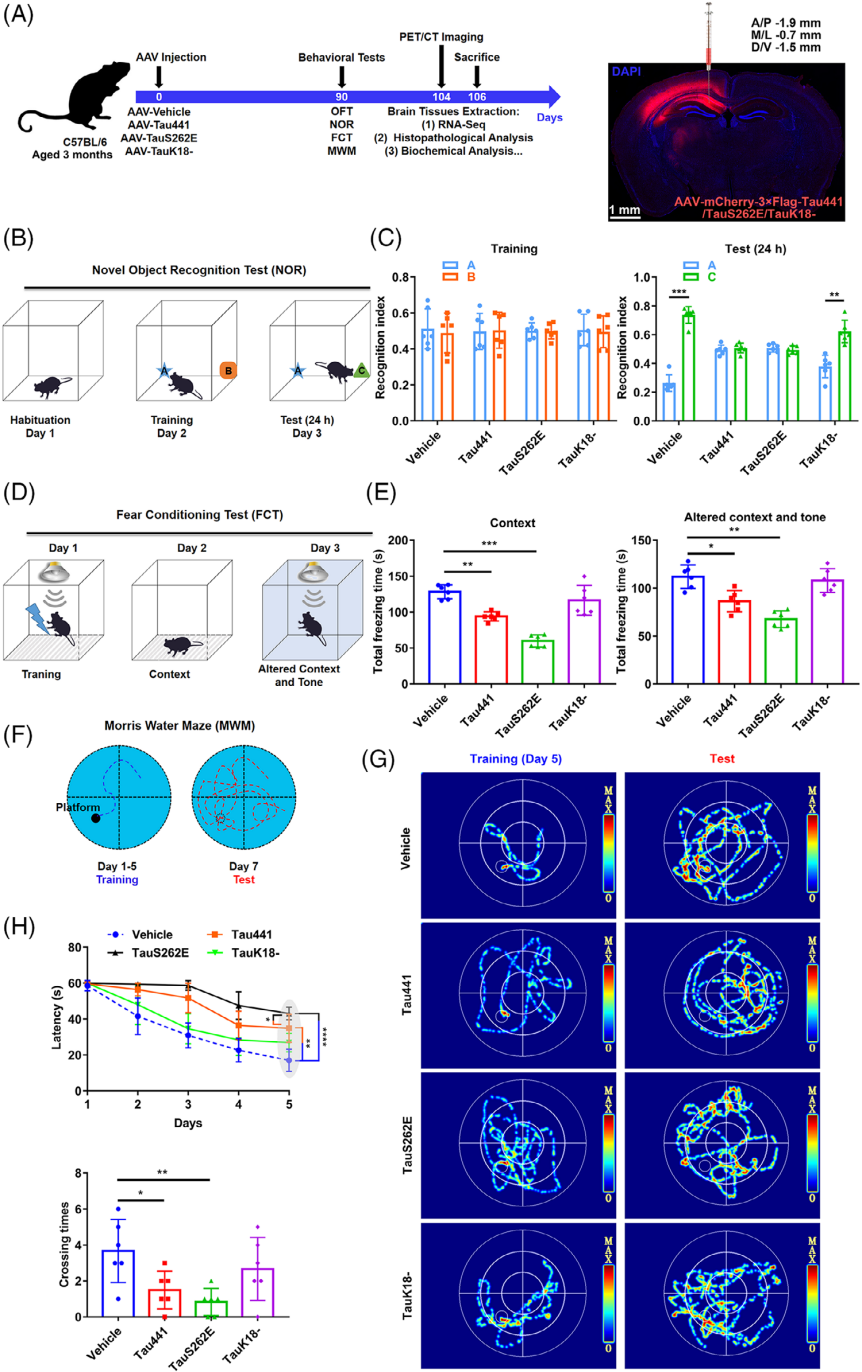
Tau overexpression in hippocampus induces cognitive impairment in C57BL/6 mice. (A) Left, animal grouping and experimental procedure. pAAV9‐hSyn‐Tau441‐mCherry‐3FLAG (Tau441 group), AAV9‐hSyn‐TauS262E‐mCherry‐3FLAG (TauS262E group), AAV9‐hSyn‐TauK18△‐mCherry‐3FLAG (TauK18– group) and control AAV (vehicle group) were injected into the left hippocampal CA1 region of 3‐month‐old C57BL/6 mice. The behaviours of the mice were evaluated by open field test (OFT), novel object recognition test (NOR), fear conditioning test (FCT) and Morris water maze test (MWM) 3 months later. Before sacrifice for brain tissue extraction, the inflammation and glucose metabolism of the brain in mice were detected via positron emission tomography/computed tomography (PET/CT) imaging. Right, the efficiency of virus infection was confirmed by fluorescence imaging. Scale bar = 1 mm. (B) The experimental design of NOR. (C) Left, recognition index for objects A and B in the acquisition trial on day 2. Right, object B was replaced by a new object C, and the recognition index for objects A and C was detected on day 3. *n* = 6 mice per group, ^**^ *p* < .01, ^***^ *p* < .001. (D) The experimental design of FCT. (E) The total freezing time (s) of the mice in the context test (left) and in the altered context and tone test (right). *n* = 6 mice per group, ^*^ *p* < .05, ^**^ *p* < .01, ^***^ *p* < .001. (F) The experimental design of MWM. (G) Representative searching trace of the training on day 5 and the probe trial at 48 h after training in MWM. (H) The latency of the mice to find the hidden platform during training on day 1 to day 5 (upper), and the times of the mice to cross the platform on day 7 (lower). *n* = 6 mice per group, ^*^ *p* < .05, ^**^ *p* < .01, ^****^ *p* < .0001 as indicated.

**FIGURE 5:**
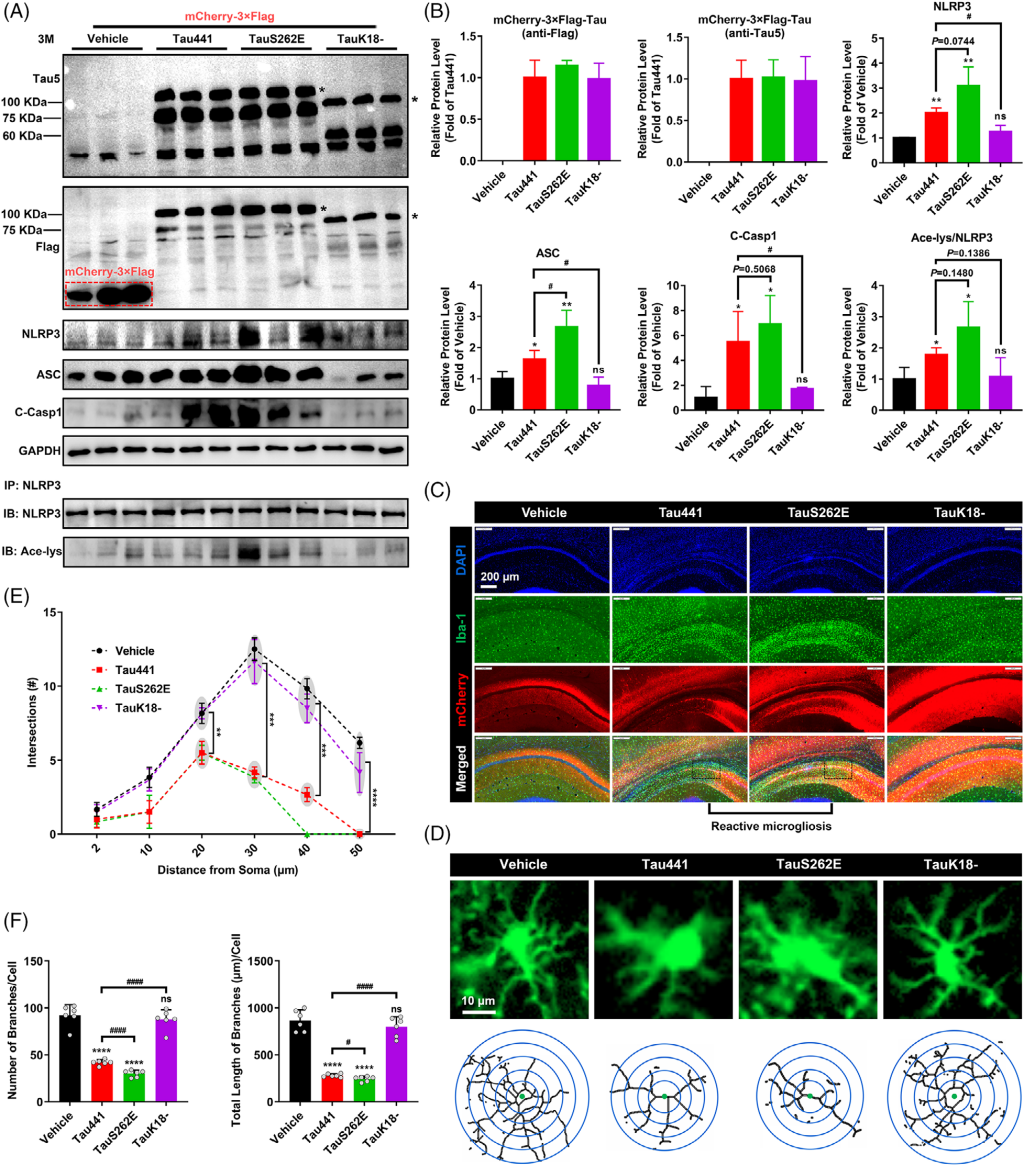
Tau overexpression promotes nucleotide‐binding oligomerisation domain (NOD)‐like receptor pyrin domain containing 3 (NLRP3) acetylation and inflammasome activation, and reactive microgliosis in mouse hippocampus. (A) Representative immunoblots of Tau5, Flag, GAPDH, NLRP3, ASC, cleaved Caspase‐1 (C‐Casp1) and acetylated NLRP3 (Ace‐NLRP3, IB: Ace‐lys/IB: NLRP3) in the hippocampi of the mice from the four groups. The target band is marked with ‘*’. (B) Quantification of the blots in (A). *n* = 3 mice per group, ns represents no significant difference, ^*^ *p* < .05, ^**^ *p* < .01 versus vehicle group, ^#^ *p* < .05. (C) Representative images of Iba‐1 immunofluorescence (green, a marker of microglia) in hippocampal region with the nuclei and overexpressed Tau proteins labelled by DAPI (blue) and mCherry (red), respectively, showing the apparent reactive microgliosis. Scale bar = 200 µm. (D) Representative fluorescence and skeleton images of single microglial cell in the four groups. Scale bar = 10 µm. (E) Sholl analysis of the microglia. Data are expressed as average number of intersections at each distance from cell bodies of all analysed cells. Two cells per animal, *n* = 3 mice per group, ^**^ *p* < .01, ^***^ *p* < .001, ^****^ *p* < .0001. (F) Quantification of the branch number and total branch length of microglia in the hippocampus. Each data point is an average value of each individual animal, three cells per animal, *n* = 6 mice per group, ns represents no significant difference, ^****^ *p* < .0001 versus vehicle group, ^#^ *p* < .05, ^####^ *p* < .0001.

To further confirm the role of Tau‐induced NLRP3 acetylation in the development of neurodegeneration in Tauopathy, we injected AAV carrying Tau441 (AAV‐Tau441), TauS262E (AAV‐TauS262E), TauK18– (AAV‐TauK18–, loss of acetyltransferase activity) or the vector control with 3×Flag‐mCherry into hippocampal CA1 region of 3‐month‐old C57BL/6 mice (Figure 4A). Three months after AAV injection, the successful overexpression of Tau proteins in hippocampus was confirmed (Figures 4A and 5A,B). Then, the cognitive function was evaluated by using NOR, MWM and FCT, respectively. In NOR, the significant memory impairment appeared in the mice of Tau441 and TauS262E‐overexpressing group, while TauK18– overexpression did not impair the visual episodic memory (Figure 4B,C). In addition, the results of FCT showed that the mice overexpressing Tau441 and TauS262E had decreased total freezing time (s) in the context and tone conditioning paradigm compared with the mice in vehicle group, indicating the fear memory impairment. Memory impairment was more pronounced in the TauS262E group than in the Tau441 group, and was unaffected in the TauK18– group (Figure 4D,E). The spatial learning and memory ability in the mice of four groups was determined by MWM. The mice in Tau441 and TauS262E group showed impaired learning ability with significantly longer latency to find the hidden platform on the fifth day of the training period. In the memory test on day 7, the number of times mice crossed the location of the platform was significantly lower in the Tau441 and TauS262E groups than in the vehicle group. However, no significant differences were observed between the TauK18– group and vehicle group during both the training and testing stages (Figure 4F–H). In addition, in OFT, there was no significant difference in the time and distance of moving among the four groups, indicating that Tau441, TauS262E and TauK18– overexpression for 3 months had no effect on the motor ability of mice (Figure S4). Taken together, these results suggest that Tau441 and TauS262E overexpression in the hippocampus for a duration of 3 months induces cognitive impairment in C57BL/6 mice.

### 3.5 Tau overexpression promotes NLRP3 acetylation and inflammasome activation, and reactive microgliosis in mouse hippocampus

As previously shown, TauK18– with deficiency of acetyltransferase activity cannot cause the activation of NLRP3 inflammasome in microglia (Figure S2I–K). Combined with the behavioural results (Figure 4), we speculate that the cognitive impairment caused by Tau441 and TauS262E overexpression may be linked to their effects on the activation of NLRP3 inflammasome in microglia and promotion of microglia activation. To verify this, we performed Western blotting to identify the changes of NLRP3 inflammasome activation related protein molecules in hippocampus. We observed elevated Caspase‐1 cleavage，and NLRP3 and ASC levels indicating NLRP3 inflammasome activation in Tau441 and TauS262E overexpression group, as well as increased NLRP3 acetylation (Figure 5A,B). IF staining of Iba‐1 and sholl analysis further confirmed that overexpression of Tau441 and TauS262E caused reactive microgliosis which was manifested by the typical activation morphology (decrease in branch length and number) and increased number of microglia in AAV‐infected brain regions (Figure 5C–F).

### 3.6 Spatial characteristics of Tauopathy and its association with activation of microglia in TauS262E overexpression model

**FIGURE 6:**
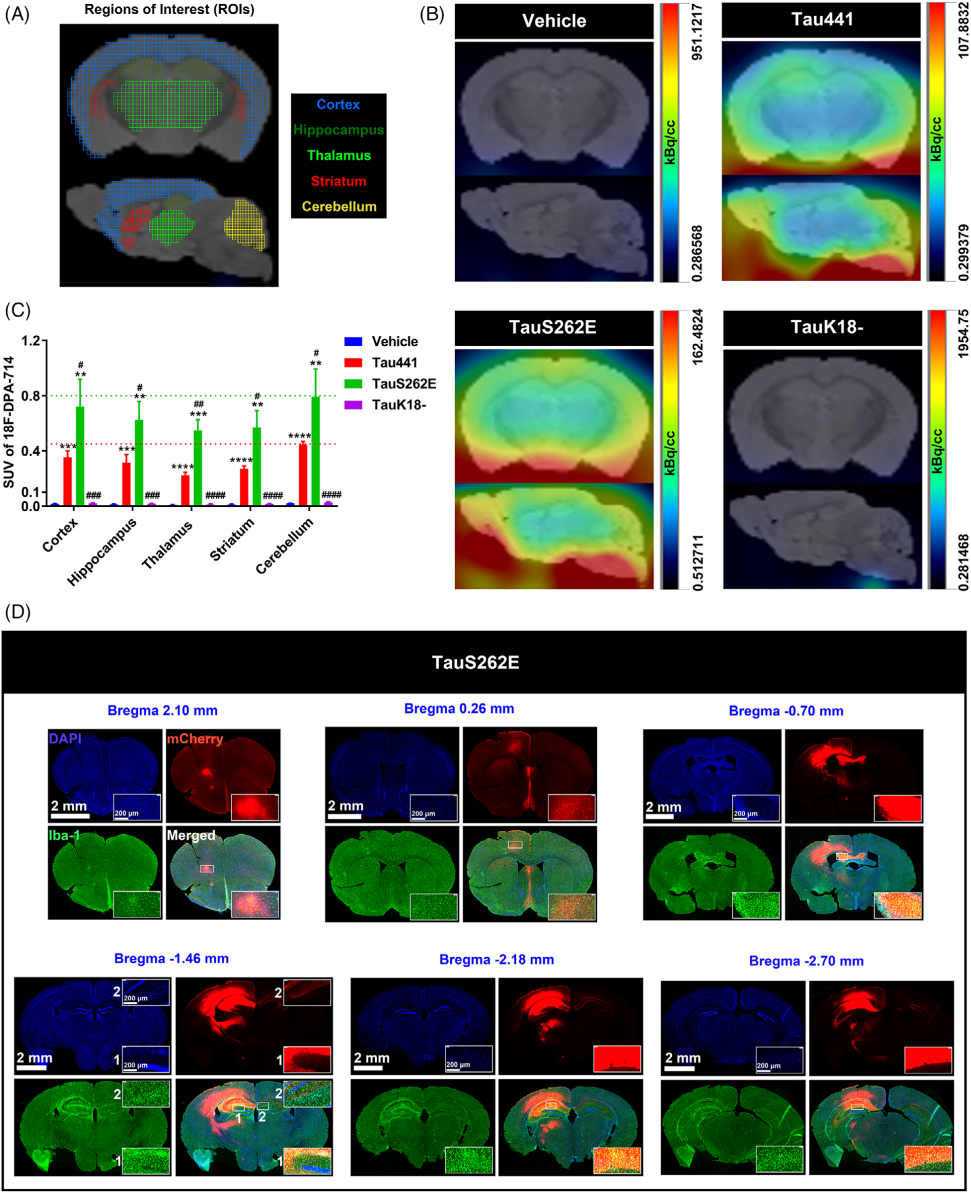
Spatial characteristics of Tauopathy and its association with activation of microglia in TauS262E overexpression model. (A) Schematic diagram of mouse brain regions of interest (coronal and sagittal section: cortex, hippocampus, thalamus, striatum and cerebellum) in positron emission tomography/computed tomography (PET/CT) imaging. (B) Representative coronal and sagittal 18F‐DPA‐714 (a ligand of the 18–kDa translocator protein [TSPO] to trace the active microglia) PET/CT images of the mice from four groups (vehicle, Tau441, TauS262E and TauK18–). (C) In vivo 18F‐DPA‐714 uptake in different brain regions of the mice in four groups is presented as standard uptake values (SUVs). *n* = 3 mice per group, ^**^ *p* < .01, ^***^ *p* < .001, ^****^ *p* < .0001 versus vehicle group, ^#^ *p* < .05, ^##^ *p* < .01, ^###^ *p* < .001, ^####^ *p* < .0001 versus Tau441 group. (D) Representative images of Iba‐1 immunofluorescence (green, a marker of microglia) in different coronal sections of TauS262E overexpressed brain with the nuclei and overexpressed TauS262E protein labelled by DAPI (blue) and mCherry (red), respectively, displaying the spatial distribution relationship between activated microglia and TauS262E protein. Scale bar = 2 mm or 200 µm.

The widespread propagation of Tau pathology is a key contributing factor for the continuous deterioration of Tauopathies.^60^ It has been demonstrated that microglia, especially in an inflammatory or reactive state, can drive Tau transmission and toxicity via various pathways.^61^, ^62^ Based on the observation that Tau acetylated NLRP3 and activated microglia, we speculated that the spatial distribution of Tau pathology would share the similar pattern with that of microglia activation. Activation of microglia leads to upregulation of the mitochondrial 18 kDa translocator protein (TSPO), which has been used as an in vivo PET imaging biomarker for neuroinflammation.^63^ To trace microglial activation in the whole brain, we performed PET/CT imaging using the radiotracer [18F]‐DPA‐714, which has been identified as a selective TSPO ligand with demonstrated good binding potential and bioavailability.^64^ As shown in Figure 6A–C, wide range of DPA‐714 high uptake signals were monitored in various regions throughout the whole brain of the mice in TauS262E‐overexpressing group, which represented the occurrence of neuroinflammation. Neuroinflammation was relatively milder in the Tau441 group than in the TauS262E group, and was almost absent in the TauK18– group, which aligns with the inability of TauK18– to induce NLRP3 acetylation. These results further confirmed the importance of NLRP3 acetylation induced by Tau in promoting microgliosis and neuroinflammation in Tauopathies. We also performed Iba‐1 IF staining on coronal sections of the entire mouse brain in the vehicle and TauS262E groups to confirm microglial activation for double validation of inflammatory response characteristics in whole brain (Figure S5A). As expected, striking microglia activation and aggregation appeared at coronal brain sections adjacent to the initiation site of AAV‐TauS262E injection (Figure 6D), while no obvious microgliosis was observed in vehicle group (Figure S5B). Furthermore, as depicted in Figures 6D and S5B, the mCherry‐labelled TauS262E proteins (red) showed a three‐dimensional, multi‐directional propagation that partly overlapped with the diffusion pattern of the AAV itself. However, through meticulous comparison, we found that the diffusion of AAV–vehicle was less pronounced particularly towards the anterior/posterior regions of the brain compared to AAV–TauS262E. Therefore, by discounting the diffusion effect of AAV, our data demonstrated that TauS262E proteins actually spread more rapidly and extensively throughout the brain compared to the virus itself. Since TauS262E potently activated microglia, this partially suggests that reactive microgliosis may reciprocally promote TauS262E propagation, which is in line with previous observations.^61^, ^62^

### 3.7 TNB peptide blocks the binding of Tau to NLRP3, and prevents NLRP3 acetylation and inflammasome activation in BV‐2 cells

**FIGURE 7:**
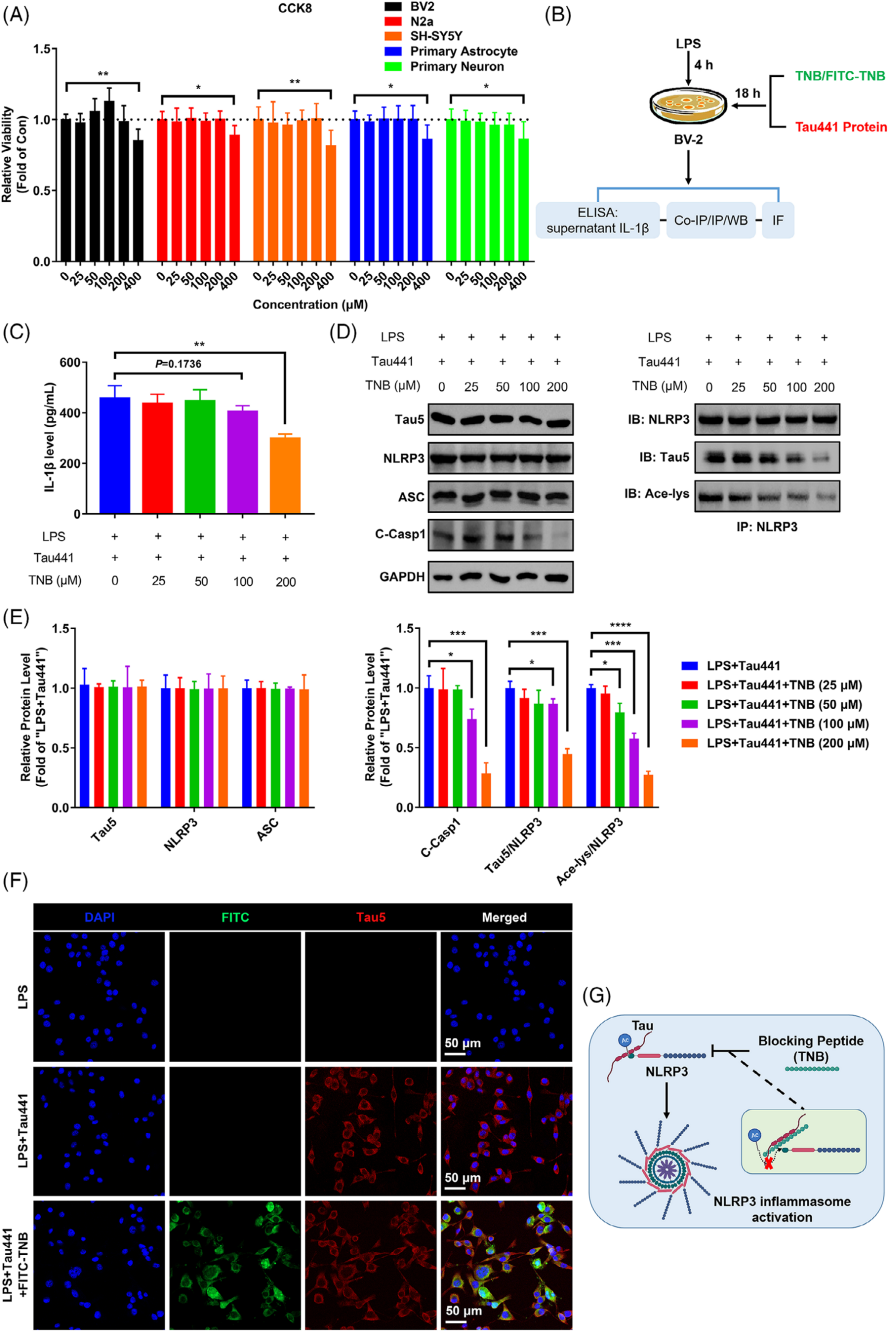
Tau–NLRP3‐binding blocking (TNB) peptide blocks the binding of Tau to nucleotide‐binding oligomerisation domain (NOD)‐like receptor pyrin domain containing 3 (NLRP3), prevents NLRP3 acetylation and inflammasome activation in BV‐2 cells. (A) CCK8 detection for the viability of different cells (BV‐2, N2a, SH‐SY5Y, primary astrocyte and primary neuron) after TNB peptide treatment at different dosages (0, 25, 50, 100, 200 and 400 µM) for 48 h. *n* = 6, ^*^ *p* < .05, ^**^ *p* < .01 as indicated. (B) Schematic diagram of in vitro experiments for testing TNB blocking function and downstream effect. (C) Enzyme‐linked immunosorbent assay (ELISA) for detecting interleukin‐1β (IL‐1β) production of LPS and Tau441 protein challenged BV‐2 microglia with or without TNB peptide treatment at different dosages (0, 25, 50, 100 and 200 µM). *n* = 3, ^**^ *p* < .01 as indicated. (D) The ability of TNB peptide of blocking Tau–NLRP3 binding and its effects on Tau‐induced NLRP3 acetylation and inflammasome activation were measured by immunoprecipitation and immunoblotting using anti‐Tau5, anti‐NLRP3, anti‐ASC, anti‐cleaved Caspase‐1 (C‐Casp1), anti‐GAPDH and anti‐acetylated‐lysine (Ace‐lys) antibody. (E) Quantification of the blots in (D). The ratio of Tau5 to NLRP3 stands for the Tau–NLRP3‐binding ability (immunoprecipitated Tau by anti‐NLRP3/NLRP3). The ratio of Ace‐lys to NLRP3 stands for the level of NLRP3 acetylation. *n* = 3, ^*^ *p* < .05, ^***^ *p* < .001, ^****^ *p* < .0001 as indicated. (F) Representative images of exogenous human Tau441 protein immunofluorescence (anti‐Tau5, red) with the nuclei and TNB peptide labelled by DAPI (blue) and FITC (green) respectively, showing the co‐localisation of TNB peptide and Tau441 protein in BV‐2 cells. Scale bar = 50 µm. (G) Mechanism diagram showing TNB peptide intercepts the binding of Tau to NLRP3, thereby inhibiting NLRP3 acetylation and preventing inflammasome activation.

To explore whether blocking NLRP3 acetylation and NLRP3 inflammasome activation induced by Tau can prevent microgliosis in Tauopathies, we designed a peptide TNB (LEDLEDVDLKKFKMHLEDYPP) based on region of NLRP3 binding with Tau to competitively inhibit Tau–NLRP3 interaction. First, we assessed the cytotoxicity of various concentrations (0, 25, 50, 100, 200 and 400 µM) of TNB on BV‐2 cells, N2a cells, SH‐SY5Y cells, primary astrocytes and primary neurons by the CCK8 assay. As shown in Figure 7A, application of TNB at concentrations lower than 200 µM did not cause any significant change in cell viability, whereas TNB at 400 µM induced significant decrease in cell viability. Therefore, we added the purified Tau441 protein and peptide TNB within a safe dose range into the BV‐2 cells pretreated with LPS (Figure 7B) in the following cell experiments. TNB could markedly inhibit IL‐1β production and Caspase‐1 cleavage induced by LPS and Tau441 protein. But the levels of NLRP3 and ASC showed no change after TNB treatment (Figure 7C–E). As expected, TNB disrupted the interaction between Tau and NLRP3 through competitive binding with Tau, consequently impeding the NLRP3 acetylation (Figure 7C–F). In addition, TNB did not interfere with Tau uptake by BV‐2 cells (Figure 7F), as alterations in this process can potentially impact NLRP3 inflammasome activation. Since we previously observed that nigericin could significantly promote NLRP3 acetylation and inflammasome activation in BV‐2 cells pretreated with LPS (Figure 2E,F), possibly in a KAT5‐dependent manner,^42^ we further verified the specificity of TNB by treating BV‐2 cells with 200 µM TNB peptide following LPS + nigericin pre‐treatment. We found that TNB could not inhibit NLRP3 acetylation and inflammasome activation induced by LPS + nigericin (Figure S6). Thus, TNB specifically and competitively binds to Tau and inhibits Tau‐mediated NLRP3 acetylation, thereby preventing inflammasome activation in BV‐2 microglia (Figure 7G).

### 3.8 Blocking Tau‐induced NLRP3 acetylation by TNB peptide rescues cognitive impairment in Tau‐overexpressing mice

**FIGURE 8:**
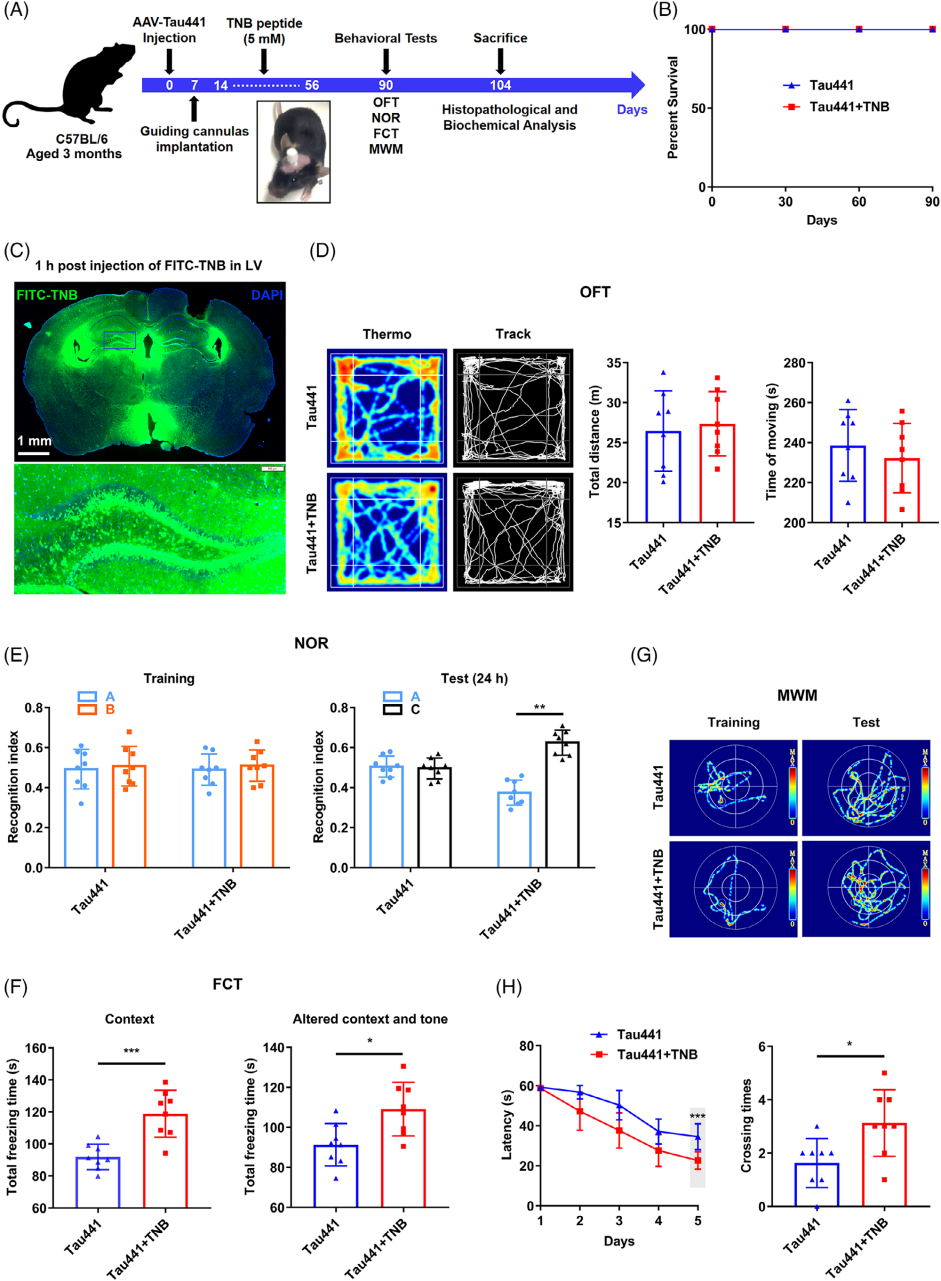
Blocking Tau‐induced nucleotide‐binding oligomerisation domain (NOD)‐like receptor pyrin domain containing 3 (NLRP3) acetylation by Tau–NLRP3‐binding blocking (TNB) peptide rescues cognitive impairment in Tau‐overexpressing mice. (A) Experimental procedure: pAAV9‐hSyn‐Tau441‐mCherry‐3FLAG was injected into the left hippocampal CA1 region of 3‐month‐old C57BL/6 mice. Two weeks later, the mice were repeatedly administered with the TNB peptide (5 mM) via left lateral ventricle‐implanted guiding cannulas once every 3 days for 6 weeks. The behaviours of the mice were evaluated by open field test (OFT), novel object recognition test (NOR), fear conditioning test (FCT) and Morris water maze test (MWM) 3 months later. Then, the mice were sacrificed for brain tissue extraction and subsequent histological and biochemical analysis. (B) Survival rate of the mice, *n* = 8 mice per group. (C) Representative images of FITC–TNB distribution in brain 1 h post‐injection of FITC–TNB in left lateral ventricle. Scale bar = 1 mm and 100 µm. (D) Representative searching trace and the total distance (m) and total time (s) of moving in OFT of the mice in two groups. *n* = 8 mice per group. (E) Left, recognition index for objects A and B in the acquisition trial in NOR. Right, object B was replaced by a new object C, and the recognition index for objects A and C was calculated 24 h later. *n* = 8 mice per group, ^**^ *p* < .01. (F) The total freezing time (s) of the mice in the context test (left) and in the altered context and tone test (right) in FCT. *n* = 8 mice per group, ^*^ *p* < .05, ^***^ *p* < .001. (G) Representative searching trace in the training on day 5 and the probe trial at 48 h after training in MWM. (H) The latency of the mice to find the hidden platform during training from day 1 to day 5 (upper), and the times of the mice to cross the platform on day 7 (lower). *n* = 8 mice per group, ^*^ *p* < .05, ^***^ *p* < .001.

**FIGURE 9:**
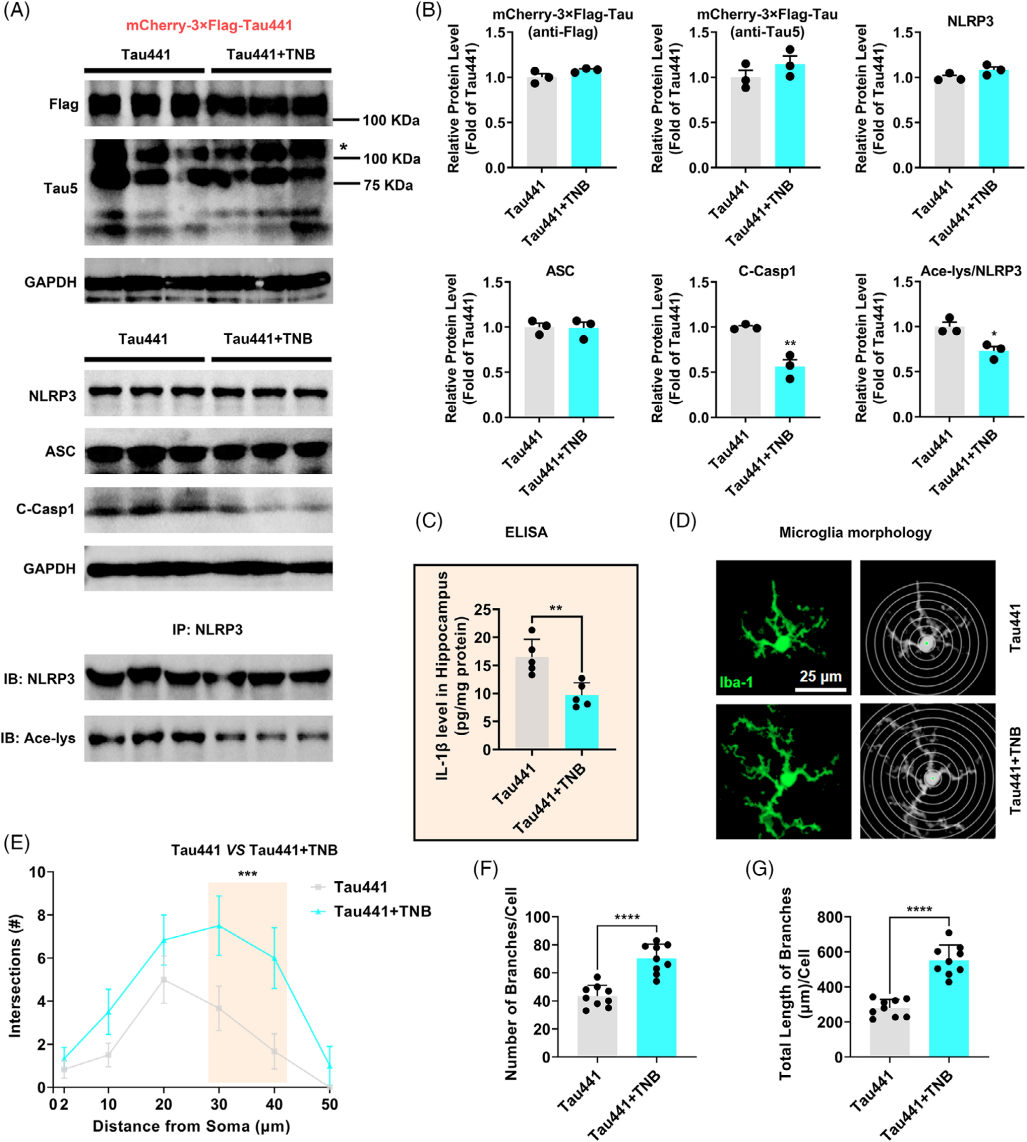
Tau–NLRP3‐binding blocking (TNB) peptide inhibits Tau‐induced nucleotide‐binding oligomerisation domain (NOD)‐like receptor pyrin domain containing 3 (NLRP3) acetylation, inflammasome activation and microglia activation in vivo. (A) Representative immunoblots of Flag, Tau5, GAPDH, NLRP3, ASC, cleaved Caspase‐1 (C‐Casp1) and acetylated NLRP3 (Ace‐NLRP3, IB: Ace‐lys/IB: NLRP3) in the hippocampi of the mice from the two groups: Tau441 and Tau441 + TNB (Tau441 overexpression for 3 months). The target band is marked with ‘*’. (B) Quantification of the blots (A). *n* = 3 mice per group, ^*^ *p* < .05, ^**^ *p* < .01 versus Tau441 group. (C) Enzyme‐linked immunosorbent assay (ELISA) detection of IL‐1β level in hippocampal tissue lysates, *n* = 5 (hippocampal tissue of five mice in each group), ^**^ *p* < .01. (D) Representative Iba‐1 staining immunofluorescence graphs of single microglia in the hippocampus region and their corresponding skeleton images. Scale Bar = 25 µm. (E) Sholl analysis of the microglia. Data are expressed as average number of intersections at each distance from cell bodies of all analysed cells. *n* = 6 (three mice in each group, with two brain slices per mouse, and one representative microglia selected from each slice), ^***^ *p* < .001. (F and G) Quantification of the branch number and total branch length of single microglia, *n* = 9 (three mice in each group, with three brain slices per mouse, and one representative microglia selected from each slice), ^****^ *p *< .0001.

To further confirm the effect of TNB peptide on Tau‐induced NLRP3 acetylation and inflammasome activation in vivo, we repeatedly administered the model mice (Tau441 overexpression in hippocampus for 3 months) with the TNB peptide (5 mM) via lateral ventricle‐implanted guiding cannulas once every 3 days for 6 weeks (Figure 8A). All mice were alive at 3 months after AAV injection (Figure 8B). We injected FITC–TNB into the lateral ventricle of C57BL/6 mice and euthanised the animals 1 h later to observe the localisation of TNB in the brain by confocal microscopy. As shown in Figure 8C, TNB was extensively distributed along the edges of several ventricles and in the hippocampus, especially in the dentate gyrus (DG). TNB was also scattered throughout the cortex and other brain regions. In the OFT, we found that Tau441 overexpression and/or TNB peptide administration did not impair motor ability of the mice (Figure 8D). TNB significantly ameliorated cognitive impairment in model mice, as indicated by increased recognition index in NOR, increased total freezing time in FCT, and shorter latency to find the hidden platform as well as increased number of platform crossings in MWM (Figure 8E–H). Consistent with the in vitro experimental results (Figure 7D,E), it was found that TNB peptide could inhibit NLRP3 acetylation and inflammasome activation in vivo without changing the levels of NLRP3 and ASC through Western blotting analysis of hippocampal samples (Figure 9A,B). Moreover, TNB peptide significantly ameliorated the activation phenotype of microglia induced by Tau (Figure 9C–G). Collectively, blocking Tau‐induced NLRP3 acetylation to inhibit inflammasome activation by TNB peptide rescues cognitive impairment in Tau‐overexpressing mice.

## 4 DISCUSSION

In the present study, we revealed a novel mechanism that Tau acetylates NLRP3 mediating inflammasome activation to activate microglia, which links Tau pathology and neuroinflammation. First, we confirmed the elevated NLRP3 inflammasome activation in the hippocampus of 3xTg‐AD mice, MAPT P301S (PS19) mice and AD patients, which is consistent with the previous studies.^26^, ^65^, ^66^ Meanwhile, we also verified that hippocampal NLRP3 acetylation level was up‐regulated in Tauopathy transgenic mice and AD patients. It has been reported that PTMs of NLRP3 such as acetylation play a vital role in regulating NLRP3 inflammasome activation.^39^, ^40^ Therefore, these results stand for a hypothesis that NLRP3 inflammasome activation in Tauopathy transgenic mice and AD patients is mediated by NLRP3 acetylation, which is partly illustrated by the high correlation between NLRP3 acetylation level and IL‐1β or cleaved Caspase‐1 level. Since Tau was identified as a bona fide acetyltransferase with enzymatic activity resides within the microtubule‐binding regions,^43^ it is thus reasonable to speculate that Tau protein may induce NLRP3 acetylation in Tauopathies. Subsequently, we found that WT or mutants mimicking pTau proteins but not K18 domain deleted Tau, which lost acetyltransferase activity significantly promoted NLRP3 acetylation and inflammasome activation in cell models. In addition, we demonstrated that Tau could directly acetylate NLRP3 at K21, K22 and K24 sites at its PYD domain and thereby induce inflammasome activation. Indeed, acetylation of NLRP3 at K21, K22 and K24 sites is critical for its full activation.^41^, ^42^ In animal experiments, overexpression of human Tau proteins in hippocampal CA1 neurons dramatically impaired the cognitive function of mice, induced NLRP3 acetylation, inflammasome activation and activated microglia. Lastly, competitive binding of TNB peptide to Tau protein inhibited its acetylation effect on NLRP3 and downstream inflammasome activation in microglia, thereby alleviating cognitive impairment. These data collectively disclose the novel mechanism of NLRP3 inflammasome activation and microgliosis in AD and related Tauopathies which is directly mediated by NLRP3 acetylation induced by pathological Tau proteins.

**FIGURE 10:**
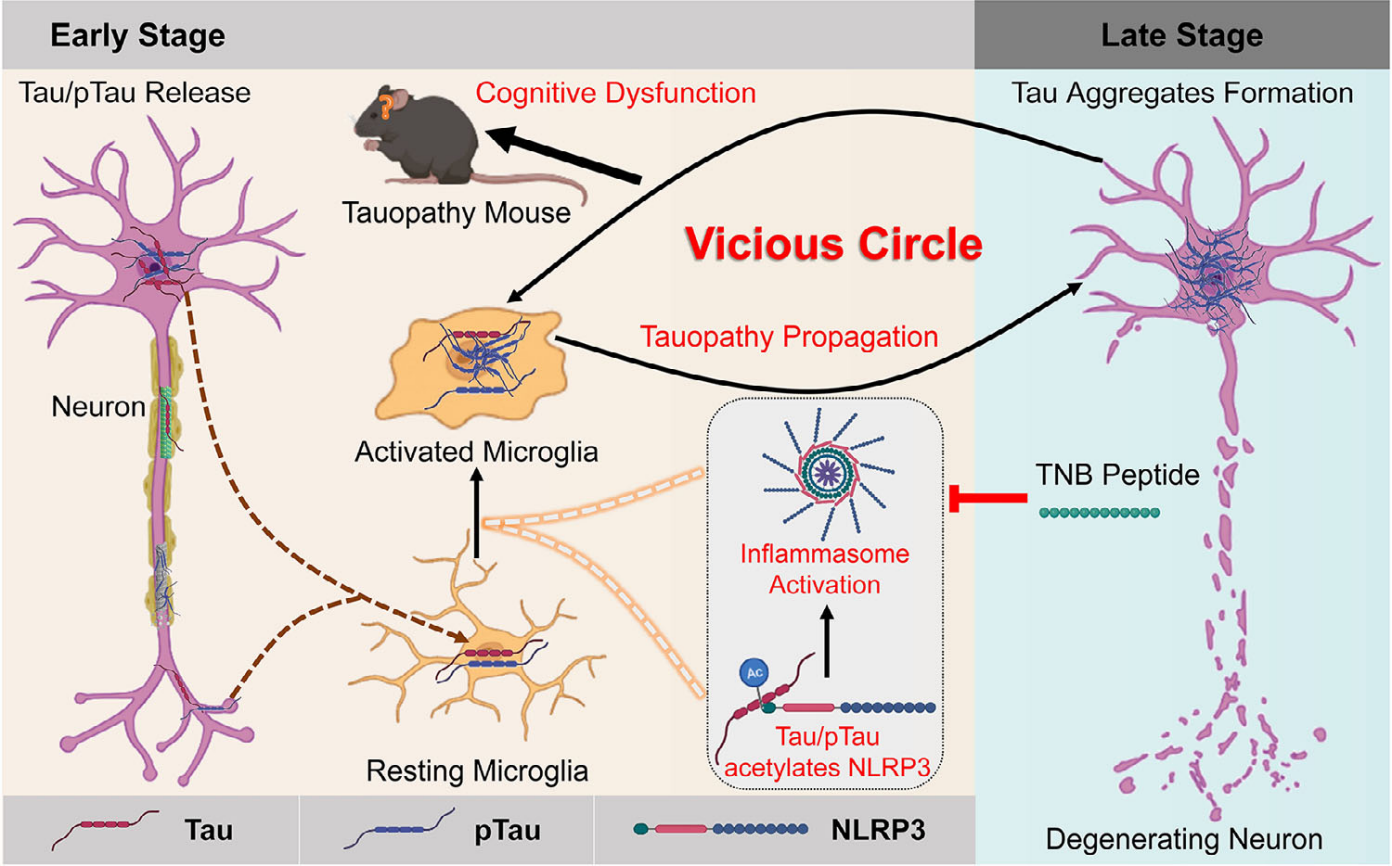
Schematic diagram of this study. At early stage of Alzheimer's disease (AD) and related Tauopathies, Tau and initial phosphorylated‐Tau (pTau) release from neurons and are sensed by microglia. Tau/pTau directly acetylates nucleotide‐binding oligomerisation domain (NOD)‐like receptor pyrin domain containing 3 (NLRP3) and induces NLRP3 inflammasome assembly and activation in microglia, thereby causing microgliosis, neuroinflammation and aggravating cognitive dysfunction. Activated microglia continuously mediating the transmission of Tauopathy to the uninfected neurons pushes the disease towards late stage. Tau aggregates formed in the late stage as damage‐associated molecular patterns (DAMPs) further enhance the activation of microglia, which forms a vicious circle. Tau–NLRP3‐binding blocking (TNB) peptide can competitively bind with Tau and effectively inhibit NLRP3 acetylation caused by Tau, thereby preventing inflammasome activation and rescuing cognitive impairment in Tauopathy mice.

Multiple studies have proved that the interactions between microglia and Tau aggregates (Tau oligomers and fibrils) in AD and related Tauopathies contribute to disease progression.^20^, ^61^, ^67^ Abnormal proteins including Tau and Aβ aggregates, act as the damage‐associated molecular patterns to initiate an inflammatory response, in which activation of NLRP3–ASC inflammasome^12^, ^19^, ^66^, ^68^ in microglial cells is a key event. NLRP3 inflammasome activation leads to cytokine IL‐1β release and initiation of inflammatory reactions.^69^ Although the study^66^ does provide a mechanistic insight into the IL‐1β priming (via MyD88‐NF‐κB) and maturation (via NLPR3 inflammasome) by different types of pathological Tau (0N3R‐T231D/S235D‐Tau), there are still unexplored steps in this causal cascade. In addition, the impact of different Tau/pTau monomers on NLRP3 inflammasome and microglia activation in the early stage of the disease remains unknown. To clarify whether Tau pathology can drive microglia‐mediated neuroinflammation during prodromal stage of Tauopathy and to uncover the related underlying mechanisms, in the present study, we selected to explore the effects of several ‘early’ pTau proteins on the activation of NLRP3 inflammasome in BV‐2 microglial cells. These ‘early’ pTau proteins including pTau‐T181, pTau‐S199, pTau‐T217 and pTau‐S262 usually increase before the development of Tau aggregation or at initial phosphorylation stage, serving as indicators for disease grading and early diagnosis.^31^ In other words, they may play a driving role in the early stage of disease to accelerate disease progression. We found that pTau‐S262 mimic, TauS262E exerted the strongest effect on NLRP3 acetylation and inflammasome activation, and the effects of other pTau proteins were slightly weaker than TauS262E but stronger than Tau441. As we known, this is the first report that Tau/pTau can directly acetylate NLRP3 and activate NLRP3 inflammasome and microglia independent on the formation of Tau aggregation. In addition, more interesting findings can be concluded from the experimental data. On the one hand, the results reflect a positive correlation between NLRP3 acetylation and inflammasome activation and verify that the acetylation ability of different Tau protein is different.^43^, ^44^, ^46^ On the other hand, because the sharp increase in pTau‐S262 occurs slightly later than other pTau proteins assessed in the present study,^31^ these data also link NLRP3 acetylation and activation of inflammasome with disease progression. Thus, it is reasonable to speculate that as the disease progresses, Tau pathology worsens with an increase in NLRP3 acetylation and inflammasome activation (Figure 1A,B). At the same time, NLRP3 inflammasome activation drives Tau pathology partially through Tau kinases in an IL‐1β‐dependent manner,^26^ which promotes the formation of a vicious cycle of ‘early’ Tau/pTau–NLRP3 acetylation/inflammasome activation‐Tau hyperphosphorylation and aggregation that continuously worsen the disease (Figure 10). As the drive factor, Tau/pTau‐induced NLRP3 acetylation is thus a valuable target for early intervention of AD and related Tauopathies.

Consistent with the findings in cell and in tube experiments, in animal experiments, TauS262E overexpression resulted in increased NLRP3 acetylation and inflammasome activation, leading to the activation phenotype of microglia and neuroinflammation; as well as significant cognitive impairment. By comparison, the weaker cognitive impairment occurred in Tau441‐overexpressing mice, and no cognitive impairment was observed in TauK18– overexpressing mice. These data established a direct contact between the degree of cognitive impairment and the acetylation level of NLRP3. Based on region of NLRP3 binding with Tau, we designed the peptide TNB containing homology sequence around K21/K22/K24 site of NLRP3. We found that TNB could competitively bind with Tau and effectively inhibited NLRP3 acetylation caused by Tau441 protein, thereby preventing inflammasome activation in BV‐2 microglia. In vivo, TNB peptide blocked Tau‐induced NLRP3 acetylation to inhibit inflammasome activation and microglia activation and then rescued cognitive impairment in Tau‐overexpressing mice. These results on the one hand further reflect the vital role of Tau‐mediated NLRP3 acetylation and inflammasome activation in the phenotypes of microglia activation and cognitive dysfunction, and also indicate that targeting NLRP3 acetylation induced by Tau is a potential promising effective strategy for early AD and related Tauopathies therapy.

In conclusion, our study not only places ‘early’ Tau pathology (pTau) upstream of microglial NLRP3 inflammasome activation in Tauopathies, but also highlights the role of Tau as an acetyltransferase in NLRP3 acetylation and provides new insight into the potential early diagnostic marker and therapeutic target for AD and related Tauopathies.

## AUTHOR CONTRIBUTIONS

Rong Liu initiated and designed this study. Rong Liu, Xiaochuan Wang and Xiaoli Lan supervised this study. Lun Zhang performed most of the molecular biological experiments and animal experiments. Lun Zhang and Yongkang Gai conducted the PEC/CT imaging. Yushuang Liu provided assistance in molecular docking. Dongli Meng, Yi Zeng, Yong Luo, Huiliang Zhang, Zhuoqun Wang, Mengzhe Yang, Yunfan Li, Yi Liu, Yiwen Lai, Shaojun Liu and Jiayu Yang assisted with animal experiments. Gang Wu designed the blocking peptide used in this study. Yu Chen, Jingtan Zhu, Tingting Yu, Ji Zeng, Dan Zhu and Jianzhi Wang provided professional guidance and technical support. Lun Zhang, Yongkang Gai and Yushuang Liu analysed the data. Lun Zhang and Rong Liu wrote the manuscript. Rong Liu, Xiaochuan Wang and Xiaoli Lan edited and reviewed the manuscript.

## CONFLICT OF INTEREST STATEMENT

The authors declare they have no conflicts of interest.

## Supporting information

Supporting Information

## Data Availability

Data will be made available on request.
