# Supplementary material for: Tau induces inflammasome activation and microgliosis through acetylating NLRP3
Source: Clin Transl Med. 2024 Mar 15;14(3):e1623. doi: 10.1002/ctm2.1623 (PMC10941548; doi:10.1002/ctm2.1623)
Supplement: Supplementary file 1 — Supporting Information [file CTM2-14-e1623-s001.docx]

**Supplementary Materials for**

**Tau induces inflammasome activation and microgliosis through acetylating NLRP3**

Lun Zhang *et al*.

Corresponding authors:

**Xiaochuan Wang** (E-mail: [wangxiaochuan@hust.edu.cn)](mailto:wxch@mails.tjmu.edu.cn));

**Xiaoli Lan** (E-mail: [xiaoli_lan@hust.edu.cn);](mailto:xiaoli_lan@hust.edu.cn);)

**Rong Liu** (E-mail: [rong.liu@hust.edu.cn](mailto:rong.liu@hust.edu.cn))

**List of Supplementary Materials**

Fig S1 to S6

Table S1**Supplementary Figures and Legends**


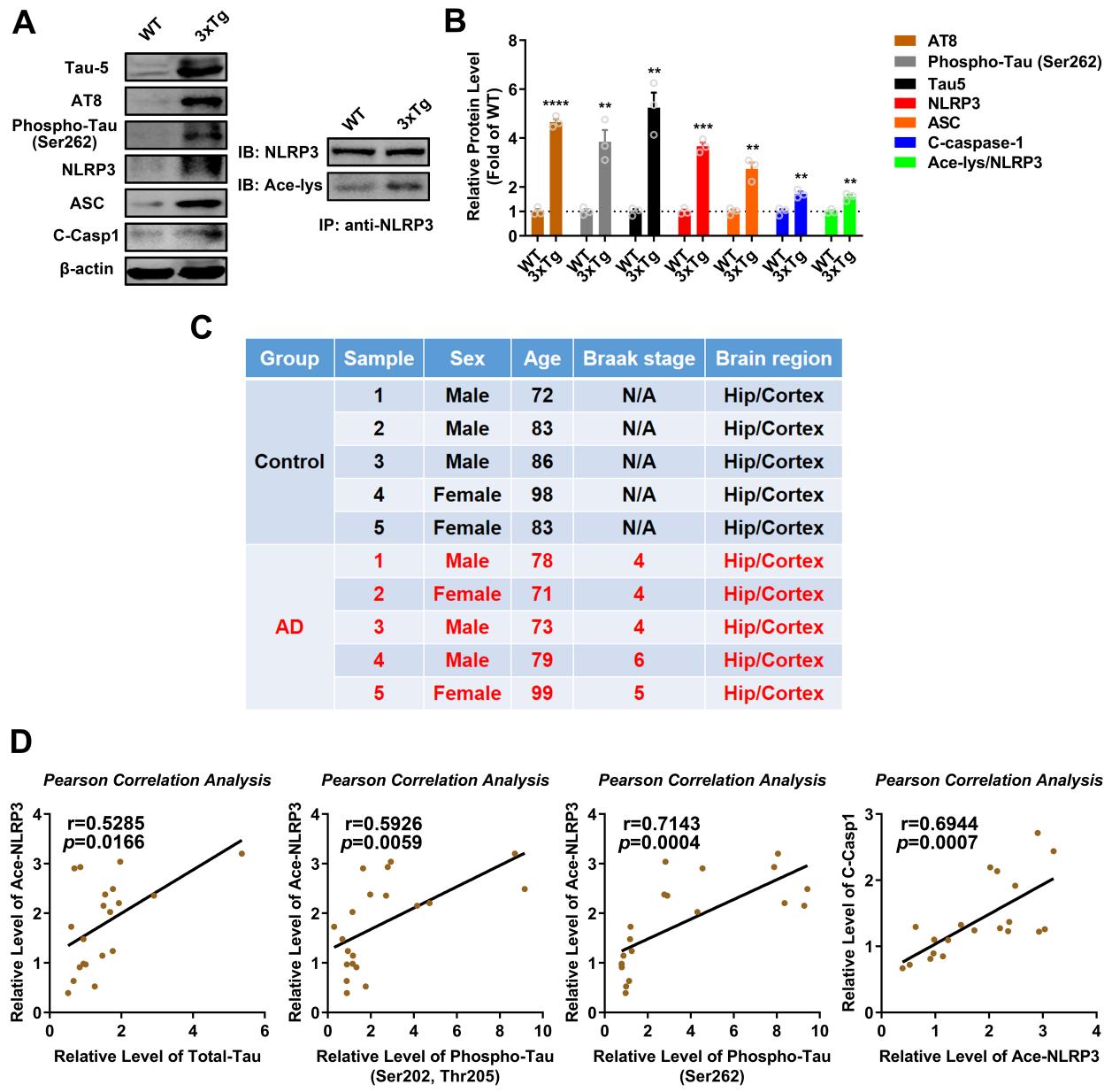


**Figure S1. (A)** Representative immunoblots of total Tau (Tau5), phosphorylated Tau (AT8, Phospho-Tau (Ser262)), NLRP3, ASC, cleaved Caspase-1 (C-Casp1) and β-actin as well as acetylated NLRP3 (Ace-NLRP3, IB: Ace-lys/IB: NLRP3) in the hippocampus of 3xTg-AD transgenic mice (9-month-old) and age matched wild-type (WT) mice. **(B)** Quantification of immunoblots in (A). n = 3, ^**^*P* < 0.01, ^***^*P* < 0.001, ^****^*P* < 0.0001 vs WT group. **(C)** Information of human brain samples. **(D)** Correlation analysis between total Tau and Ace-NLRP3 (r = 0.5285, *p* = 0.0166), between phosphorylated Tau (AT8) and Ace-NLRP3 (r = 0.5926, *p* = 0.0059), between phosphorylated Tau (Phospho-Tau (Ser262)) and Ace-NLRP3 (r = 0.7143, *p* = 0.0004) and between Ace-NLRP3 and C-Casp1 (r = 0.6944, *p* = 0.0007) in Control/AD patients based on the data from Fig. 1 (D) and (E).

**
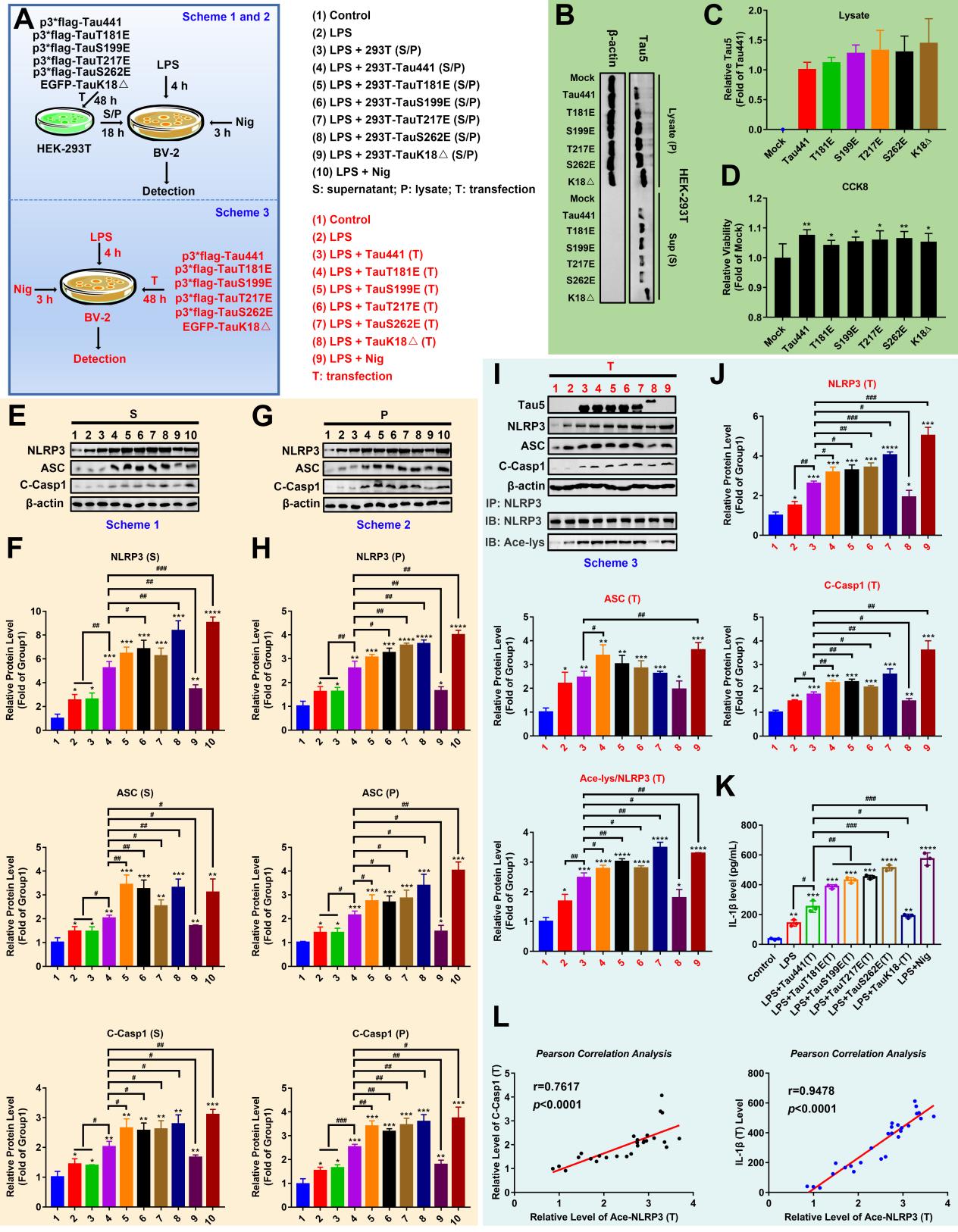
**

**Figure S2. Tau proteins activate NLRP3 inflammasome in three cell models. (A)** Schematic diagram of the three cell models for effect evaluation of different Tau proteins on NLRP3 acetylation and inflammasome activation. Scheme 1 and 2: after 4 h pretreatment with 1 μg/mL LPS, BV-2 microglial cells were incubated with the culture media (supernatant)/cell lysate (S/P) from HEK-293T cells transfected with/without Tau (Tau441, TauT181E, TauS199E, TauT217E, TauS262E and TauK18Δ) for 18 h, or nigericin (Nig, 20 μM, positive control) for 3 h. Then the NLRP3 inflammasome activation in BV-2 cells was detected by Western blotting. Scheme 3: after 4 h pretreatment with 1 μg/mL LPS, BV-2 cells were overexpressed different Tau proteins same as in Scheme 1 and 2 for 48 h, then the NLRP3 acetylation and inflammasome activation were detected by immunoprecipitation and immunoblotting. **(B)** Representative immunoblots of Tau protein (Tau5) and β-actin in culture media and cell lysate of HEK-293T cells overexpressing different Tau proteins, showing the successful Tau expression and release. **(C)** Quantification of Tau5 in cell lysates. n = 3. **(D)** CCK8 assay for evaluation of the viability of HEK-293T cells overexpressing different Tau proteins. n = 12 in Mock group, n = 6 in the other groups. ^*^*P* < 0.05, ^**^*P* < 0.01 vs Mock group. **(E-H)** Representative immunoblots and quantification of NLRP3, ASC, cleaved Caspase-1 (C-Casp1) and β-actin in scheme 1 (E and F) and scheme 2 (G and H). The groups are numbered from 1 to 10 and highlighted in black as shown in (A). n = 3, ^*^*P* < 0.05, ^**^*P* < 0.01, ^***^*P* < 0.001, ^****^*P* < 0.0001 vs Group 1 (Control group), ^#^*P* < 0.05, ^##^*P* < 0.01, ^###^*P* < 0.001 as indicated. **(I-J)** Representative immunoblots (I) and quantification (J) of transfected Tau (Tau5, quantification data not shown), NLRP3, ASC, C-Casp1, β-actin and acetylated NLRP3 (Ace-NLRP3, IB: Ace-lys/IB: NLRP3) in scheme 3. The groups are numbered from 1 to 9 and highlighted in red as shown in (A). n = 3, ^*^*P* < 0.05, ^**^*P* < 0.01, ^***^*P* < 0.001, ^****^*P* < 0.0001 vs Group 1 (Control group), ^#^*P* < 0.05, ^##^*P* < 0.01, ^###^*P* < 0.001 as indicated. **(K)** ELISA assay of the IL-1β production by BV-2 cells in scheme 3. n = 3, ^**^*P* < 0.01, ^***^*P* < 0.001, ^****^*P* < 0.0001 vs Control group, ^#^*P* < 0.05, ^##^*P* < 0.01, ^###^*P* < 0.001 as indicated. **(L)** Correlation analysis between Ace-NLRP3 and C-Casp1 (r = 0.7617, *p* < 0.0001), and between Ace-NLRP3 and IL-1β (r = 0.9478, *p* < 0.0001) based on the data from (I) to (K).


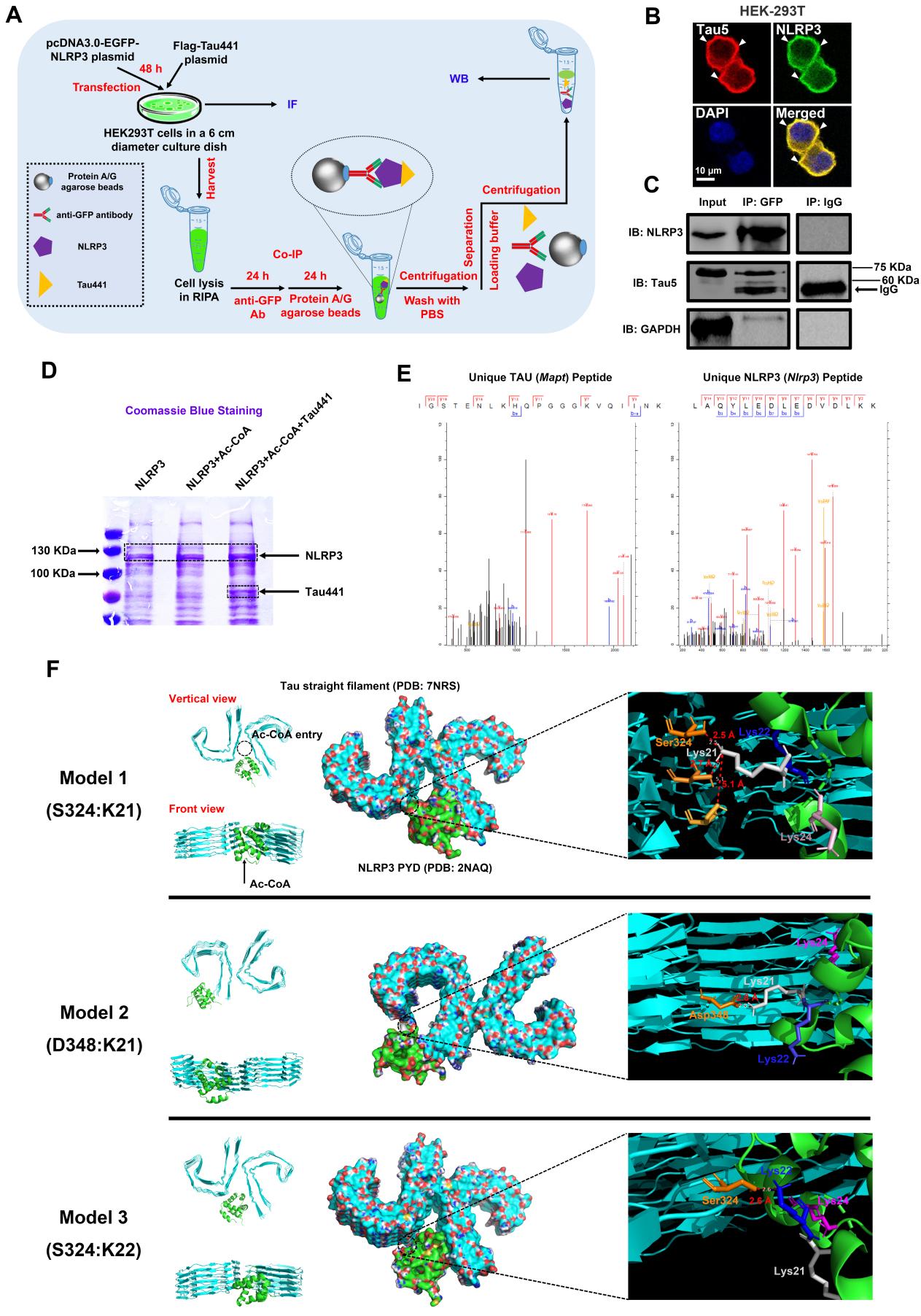


**Figure S3. (A)** Schematic diagram of the detection of Tau and NLRP3 interaction in HEK-293T cells. HEK-293T cells were co-transfected with EGFP-NLRP3 (fusion with EGFP) and Flag-Tau441 plasmid for 48 h, immunofluorescence staining was conducted for the localization of Tau and NLRP3, or NLRP3 was immunoprecipitated by anti-GFP antibody to confirm whether there was an interaction of NLRP3 with Tau through Western blotting. **(B)** Representative images of Tau5 (red) and NLRP3 (green) immunofluorescence, showing co-localization of Tau and NLRP3. Scale bar = 10 μm. **(C)** Representative immunoblots of Tau protein (Tau5) and NLRP3 showing the binding of NLRP3 with Tau441 protein. **(D)** Coomassie blue staining shows the products of acetylation assay in test-tube in Fig. 3. **(E)** Mass spectrometry analysis of NLRP3-interacting proteins. The Tau and NLRP3 typical mass spectrometry peptide spectra are displayed. **(F)** Visualization of molecular docking models of NLRP3-PYD domain (PDB: 2NAQ) and Tau straight filament (PDB: 7NRS) with low energy.


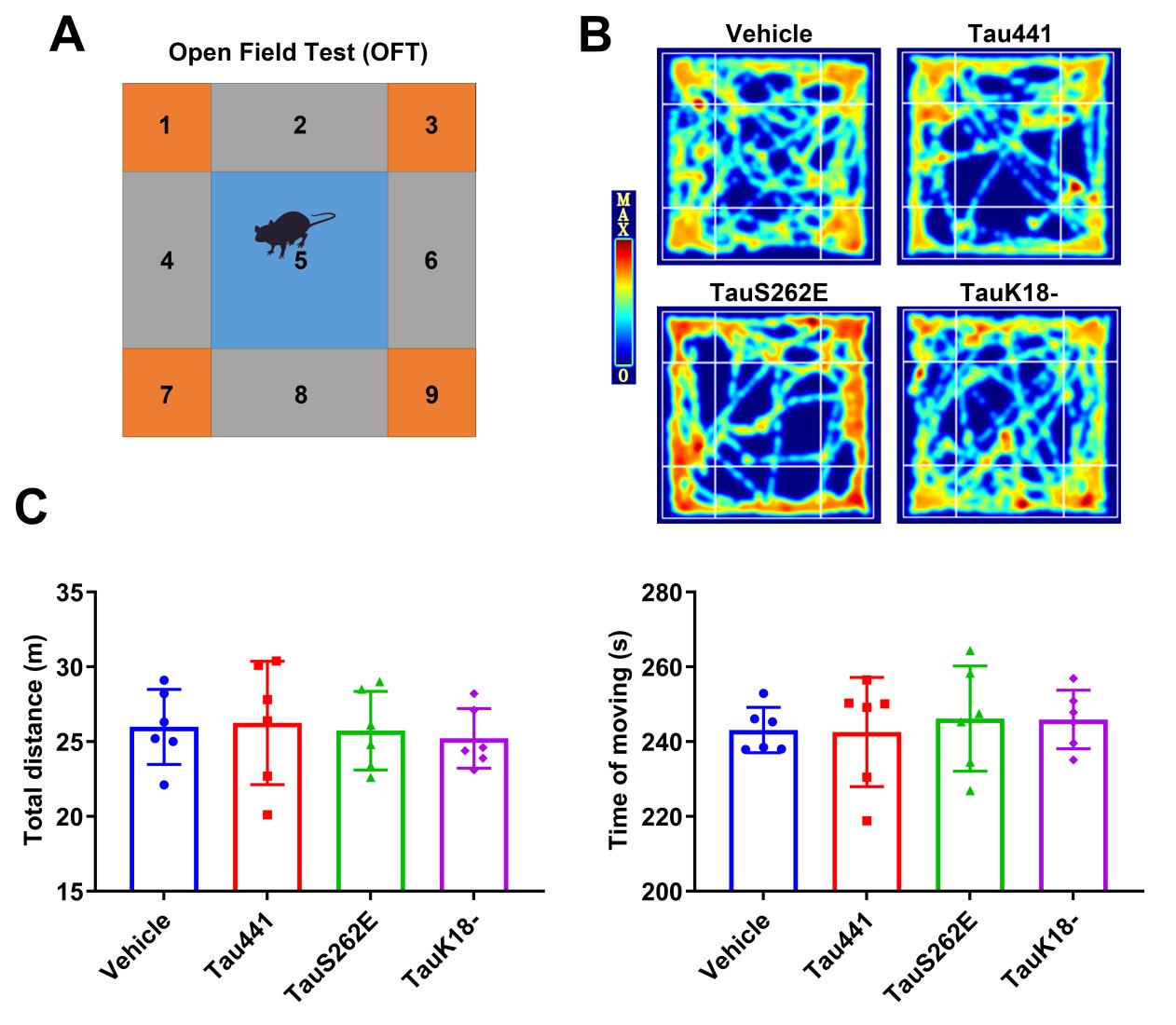


**Figure S4. Tau overexpression does not induce motor ability deficit in mice. (A)** The experimental design of OFT: the zone 5 serves as the centre. **(B)** Representative traces in the field. **(C)** The total distance (m) and total time (s) of moving. n = 6 mice for each group.


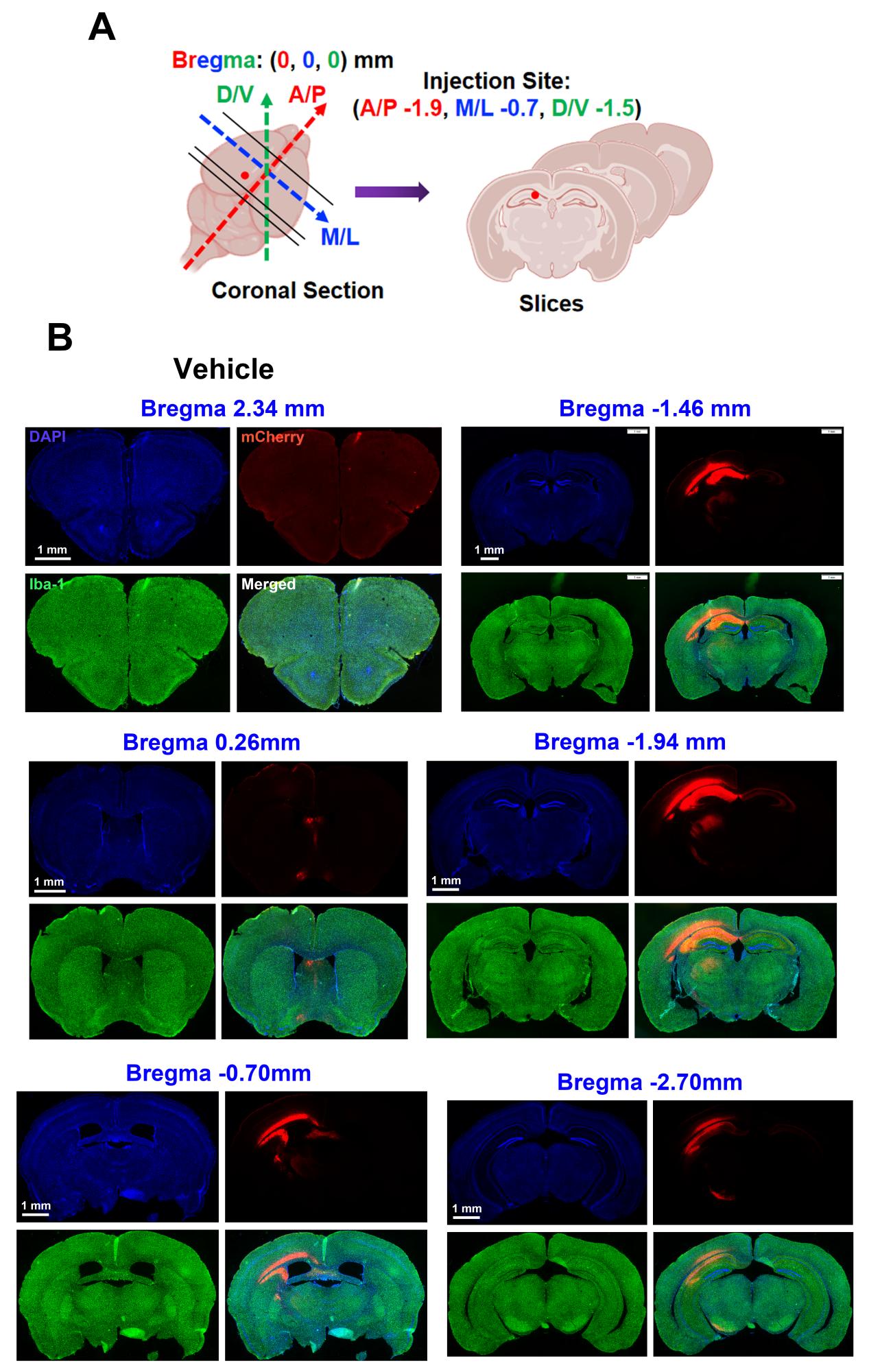


**Figure S5.** **(A)** Schematic diagram of continuous coronal sectioning for Iba-1 immunostaining. **(B)** Representative images of Iba-1 immunofluorescence (green, a marker of microglia) in different coronal sections of the brain from Vehicle group with the nuclei and control vector labeled by DAPI (blue) and mCherry (red) respectively, showing no obvious activation of microglia in the whole brain. Scale bar = 1 mm.


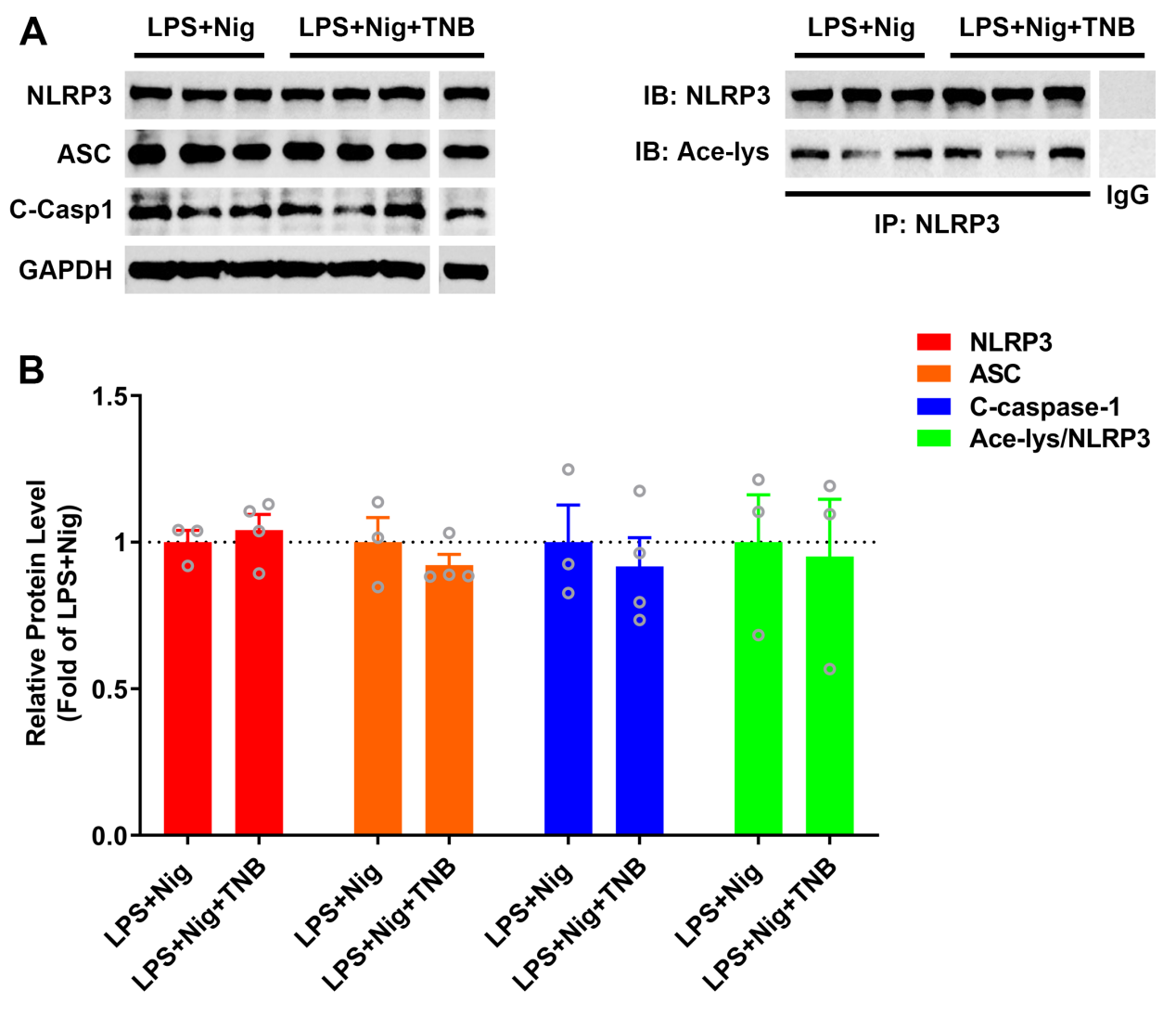


**Figure S6.** **TNB peptide could not prevent NLRP3 acetylation and inflammasome activation in BV-2 cells challenged by LPS and nigericin.** **(A)** The effects of TNB peptide (200 μM) on LPS+nigericin-induced NLRP3 acetylation and inflammasome activation were measured by immunoprecipitation and immunoblotting using anti-NLRP3, anti-ASC, anti-cleaved Caspase-1 (C-Casp1), anti-GAPDH and anti-acetylated-lysine (Ace-lys) antibody. **(B)** Quantification of the blots in (A). The ratio of Ace-lys to NLRP3 stands for the level of NLRP3 acetylation. n = 3 or 4.

**Table S1** The primary antibodies used in this study

| Antibody | Type | Dilution | Source |
| --- | --- | --- | --- |
| Tau-5 | Mouse mAb | WB: 1:1000 ICC: 1:200 | Abcam (Cat# ab80579) |
| NLRP3 | Rabbit mAb | WB: 1:1000 IP: 1:200 | CST (Cat# 15101) |
| NLRP3 | Rabbit pAb | ICC: 1:50 | ZENBIO (Cat# 381207) |
| ASC | Rabbit pAb | WB: 1:1000 | ZENBIO (Cat# 340097) |
| Cleaved Caspase-1 | Rabbit mAb | WB: 1:1000 | CST (Cat# 89332) |
| Cleaved Caspase-1 | Rabbit pAb | WB: 1:500 | ZENBIO (Cat# 341030) |
| Caspase-1 | Rabbit pAb | WB: 1:500 | ZENBIO (Cat# 342947) |
| Sirt2 | Rabbit pAb | WB: 1:1000 | Proteintech (Cat# 19655-1-AP) |
| AT-8 | Mouse mAb | WB: 1:1000 | ThermoScientific (Cat# MN1020) |
| Phospho-Tau (Ser262) | Rabbit pAb | WB: 1:1000 | ZENBIO (Cat# 310195) |
| GAPDH | Rabbit pAb | WB: 1:1000 | ZENBIO (Cat# 380626) |
| β-actin | Mouse mAb | WB: 1:5000 | ThermoScientific (Cat# 66009-1-IG) |
| Acetylated-Lysine (Ace-lys) | Rabbit pAb | WB: 1:1000 | CST (Cat# 9441S) |
| Iba-1 | Rabbit pAb | IF: 1:500 | Wako (Cat# 019-19741) |
| Flag | Mouse mAb | WB: 1:1000 IP: 1:20 | ZENBIO (Cat# 250111) |
| GFP | Mouse mAb | IP: 1:20 | ZENBIO (Cat# 250065) |
